# The ventrolateral medulla and medullary raphe in sudden unexpected death in epilepsy

**DOI:** 10.1093/brain/awy078

**Published:** 2018-03-28

**Authors:** Smriti Patodia, Alyma Somani, Megan O’Hare, Ranjana Venkateswaran, Joan Liu, Zuzanna Michalak, Matthew Ellis, Ingrid E Scheffer, Beate Diehl, Sanjay M Sisodiya, Maria Thom

**Affiliations:** 1 Departments of Neuropathology, UCL, Institute of Neurology, Queen Square, London WC1N 3BG, UK; 2 Clinical and Experimental Epilepsy and Chalfont Centre for Epilepsy, UCL, Institute of Neurology, Queen Square, London WC1N 3BG, UK; 3 Department of Biomedical Sciences, University of Westminster London W1W 6UW, UK; 4 Epilepsy Research Centre, Department of Medicine (Neurology), University of Melbourne, Victoria 3052, Australia

**Keywords:** SUDEP, brainstem, pre-Bötzinger complex, medullary raphe, stereology

## Abstract

Sudden unexpected death in epilepsy (SUDEP) is a leading cause of premature death in patients with epilepsy. One hypothesis proposes that sudden death is mediated by post-ictal central respiratory depression, which could relate to underlying pathology in key respiratory nuclei and/or their neuromodulators. Our aim was to investigate neuronal populations in the ventrolateral medulla (which includes the putative human pre-Bötzinger complex) and the medullary raphe. Forty brainstems were studied comprising four groups: 14 SUDEP, six epilepsy controls, seven Dravet syndrome cases and 13 non-epilepsy controls. Serial sections through the medulla (from obex 1 to 10 mm) were stained for Nissl, somatostatin, neurokinin 1 receptor (for pre-Bötzinger complex neurons) and galanin, tryptophan hydroxylase and serotonin transporter (neuromodulatory systems). Using stereology total neuronal number and densities, with respect to obex level, were measured. Whole slide scanning image analysis was used to quantify immunolabelling indices as well as co-localization between markers. Significant findings included reduction in somatostatin neurons and neurokinin 1 receptor labelling in the ventrolateral medulla in sudden death in epilepsy compared to controls (*P* < 0.05). Galanin and tryptophan hydroxylase labelling was also reduced in sudden death cases and more significantly in the ventrolateral medulla region than the raphe (*P* < 0.005 and *P* < 0.05). With serotonin transporter, reduction in labelling in cases of sudden death in epilepsy was noted only in the raphe (*P* ≤ 0.01); however, co-localization with tryptophan hydroxylase was significantly reduced in the ventrolateral medulla. Epilepsy controls and cases with Dravet syndrome showed less significant alterations with differences from non-epilepsy controls noted only for somatostatin in the ventrolateral medulla (*P* < 0.05). Variations in labelling with respect to obex level were noted of potential relevance to the rostro-caudal organization of respiratory nuclear groups, including tryptophan hydroxylase, where the greatest statistical difference noted between all epilepsy cases and controls was at obex 9–10 mm (*P* = 0.034), the putative level of the pre-Bötzinger complex. Furthermore, there was evidence for variation with duration of epilepsy for somatostatin and neurokinin 1 receptor. Our findings suggest alteration to neuronal populations in the medulla in SUDEP with evidence for greater reduction in neuromodulatory neuropeptidergic and mono-aminergic systems, including for galanin, and serotonin. Other nuclei need to be investigated to evaluate if this is part of more widespread brainstem pathology. Our findings could be a result of previous seizures and may represent a pathological risk factor for SUDEP through impaired respiratory homeostasis during a seizure.

## Teaser

Pathological changes in brainstem respiratory nuclei may underlie SUDEP. In this post-mortem study, Patodia *et al*. reveal alterations in pre-Bötzinger region neurons and modulatory medullary neuropeptidergic and monoaminergic systems, including galanin, somatostatin and serotonin. These changes may be sequelae of seizures and risk factors for SUDEP through defective respiratory homeostasis.

## Introduction

Sudden and unexpected death in epilepsy (SUDEP) is the leading cause of death in young adults with intractable epilepsy (Dlouhy *et al.*, 2016). Ictal respiratory dysfunction occurs in a subset of patients with epilepsy (Kennedy and Seyal, 2015) and has been observed as a terminal event in witnessed SUDEP cases on epilepsy monitoring units (Ryvlin *et al.*, 2013). There is accumulating evidence from clinical and experimental models that a centrally mediated apnoea could underlie some SUDEP cases (Sowers *et al.*, 2013; Dlouhy *et al.*, 2016).

Central control of respiration is through interconnected medullary nuclear groups forming the ventral respiratory column (Smith *et al.*, 2013) in the ventrolateral medulla (VLM). Largely based on findings in animal studies, these nuclei include the pre-Bötzinger complex (pre-BötC), an essential generator of inspiratory rhythm (McKay *et al.*, 2005; Feldman and Del Negro, 2006), comprising diverse neuronal cell types, including pacemaker-like somatostatin (SST) and neurokinin 1 receptor (NK1R)-positive cells, as well as glycinergic and GABAergic interneurons (Stornetta *et al.*, 2003; Ramirez-Jarquin *et al.*, 2012; Wei *et al.*, 2012). The pre-BötC is further modulated to regulate respiratory rhythm in response to physiological and metabolic demands and during sleep-wake cycles via higher cortical and other brainstem nuclei (Ramirez *et al.*, 2012; Smith *et al.*, 2013). The latter are regulated by peripheral and sensory inputs and chemoreceptors and, through the serotonergic neurons of the medullary raphe, provide excitatory drive in response to hypoxia and hypercarbia (Richerson, 2004; Benarroch, 2014). Emerging data from clinical and experimental studies implicate defective serotonergic systems in the respiratory dysfunction and impaired arousal following seizures that could be relevant to SUDEP (Richerson, 2013; Sowers *et al.*, 2013; Zhan *et al.*, 2016). In sudden infant death syndrome (SIDS), which has clinical parallels with SUDEP, alterations to medullary serotonergic neuronal populations have been shown (Paterson *et al.*, 2006; Kinney *et al.*, 2009).

In an MRI study of SUDEP, observed volume loss in the medulla was proposed to be secondary to sustained seizure propagation in this region and relevant to ictal autonomic disturbances in SUDEP (Mueller *et al.*, 2014). Spreading depolarizations in the dorsal medulla following seizures mediate cardio-respiratory arrest in mouse SUDEP models (Aiba and Noebels, 2015). Previous post-mortem studies in neurodegenerative conditions such as multiple system atrophy and Parkinson’s disease, with associated sleep-related respiratory disorders and sudden death, show pathology in both the pre-BötC and the medullary raphe (Tada *et al.*, 2009; Schwarzacher *et al.*, 2011; Presti *et al.*, 2014), but these regions remain unexplored in SUDEP.

We hypothesized that pathological changes in brainstem respiratory nuclei could occur in SUDEP and our aim was to study the VLM region, which encloses the putative human homologue of the pre-BötC nucleus (Tada *et al.*, 2009; Schwarzacher *et al.*, 2011; Presti *et al.*, 2014) and medullary raphe in a series of SUDEP post-mortem cases compared with control groups.

## Materials and methods

### Case selection

Brainstems from 40 post-mortem cases were included in this study. Tissue from all cases was retained with era-appropriate consent. Cases included:

- Fourteen SUDEP from the Epilepsy Society Brain and Tissue Bank (ESBTB) at UCL (collected between 2010 and 2015) and from *Brain UK* via the pathology department at Derriford Hospital, Plymouth (between 2007 and 2012). These were further categorized into nine definite SUDEP (complete and negative autopsy including toxicology), the remaining five being probable or possible SUDEP (incomplete autopsy examination or competing cause of death identified) (Nashef *et al.*, 2012);
- Seven cases with Dravet syndrome obtained from ESBTB and the University of Melbourne Australia [as previously reported (Catarino *et al.*, 2011), between 1992 and 2010]. This group also included three SUDEP cases (two of which were definite SUDEP);
- Thirteen non-epilepsy controls were obtained through the MRC Sudden Death Brain Bank, Edinburgh and ESBTB (between 2008 and 2015). These included 10 cases with sudden death (non-neurological, non-epilepsy sudden death controls);
- Six epilepsy controls without an epilepsy-related death (1999–2015). The clinical and neuropathology records, including epilepsy and drug history, chronicity, circumstances of death and main neuropathology findings are detailed in Supplementary Table 1 and summarized in Table 1. Of note, in all epilepsy groups there were cases with onset of seizures in the last 2 years of life (3/14 in SUDEP, 1/7 in Dravet syndrome and 3/6 in epilepsy controls) as well as cases with epilepsy duration of >10 years (8/14 in SUDEP, 5/7 in Dravet syndrome and 3/6 in epilepsy controls).

**Table 1:** Summary of clinical details for the 40 cases in the four main groups studied

Group	*n*	Gender M/F	Mean age onset of epilepsy/mean duration (years)	Mean age of death, years (range)	Mean brain weight^a^, g (range)	Mean mid obex level, mm (range)	PMI/FT mean (days)
**SUDEP**							
All (non-DS SUDEP)	14	8/6	13.6 /19	35.4 (18–53)	1399 (1310–1623)	6 (2–8)	3.2 /31
D-SUDEP	9	4/5	13.1	34.6	1365	7.3	
P-SUDEP	5	4/1	14.6	36.8	1459	6.5	
**Dravet syndrome**							
ALL	7	4/3	0.8/18	18.7 (1–47)	1189 (1078–1340)	7 (4–13)	1.2/48
P-SUDEP	1	2/0	0.78	24	1151	10.5	
D-SUDEP	2	1/0	0.6	11	1300	4	
**Non-epilepsy controls**							
All	13	10/3	NA	41.5 (23–80)	1469 (1374–1650)	6.5 (3.5–11.5)	3.4/15
NESD	10	8/2	NA	38.7	1469	6.8	
**Epilepsy controls**	6	5/1	27/43	67 (47–84)	1307 (1185–1490)	8 (4–10.5)	2.3/50

Detailed case information is provided in Supplementary Table 1. From these four groups there were further subdivisions for definite SUDEP (D-SUDEP), possible or probable SUDEP (P-SUDEP), and non-epilepsy sudden deaths (NESD).

^a^Mean brain weights are given for the fresh weights; where the fresh weights were not available and only fixed weights 22 g was subtracted (based on previous study of brain weights in SUDEP) (Thom *et al.*, 2015). There was no significant difference in post-mortem interval between SUDEP epilepsy controls and non-epilepsy control groups.

DS = Dravet syndrome; FT = fixation times; NA = not applicable; PMI = post-mortem interval.

### Tissue preparation

For all 40 cases, a single 5-mm thick medulla block was selected. Where available, blocks were selected from the caudal medulla (axial level between obex 0 and 12 mm); in many cases only one block of medulla was available for use. In seven cases, only hemi-brainstems were used as the brainstem had been divided sagittally as part of a protocol for 9.4 T MRI brainstem imaging (Patodia *et al.*, in preparation) but all these cases included the entire midline raphe nuclei. Serial sections were cut through the block at 20-μm thickness using the Tissue-Tek AutoSection automated microtome (Sakura Finetek) obtaining 150–200 sections per case. Every 10th section (equivalent to ∼200 μm steps) was stained for cresyl violet and obex levels were confirmed independently by two observers (M.T., S.P.) using a standard atlas (Paxinos and Huang, 1995). A region spanning 10 consecutive cresyl violet levels (equivalent to 2 mm rostro-caudally) in each case was selected for further quantitative analysis, closest to the putative location of the pre-BötC as based on human anatomical studies (Schwarzacher *et al.*, 2011). The mean mid-obex level for all cases was 6.5 mm (range 2–13 mm) (Table 1) and there was no statistical difference in mean mid-obex levels between the groups.

### Immunohistochemistry and regions of interest

**Figure 1:**
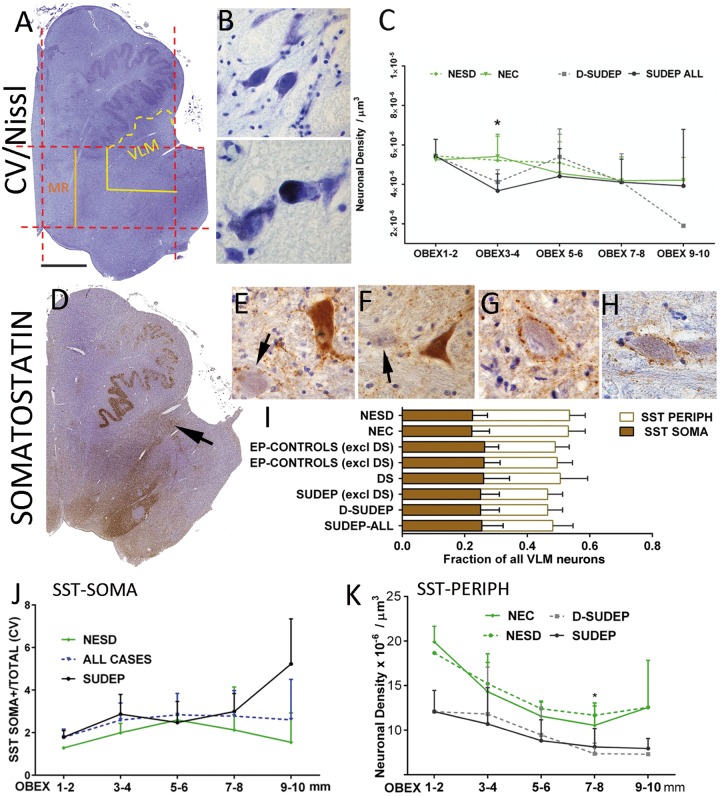
**Stereology analysis of neurons in VLM.** (**A**) Cresyl violet (CV). The regions of interest were delineated using the image analysis systems by drawing a rectangle in one-half of the brainstem (dashed red lines) using anatomical boundaries. The midline was first drawn and a parallel line at the lateral edge of the inferior olive nucleus. Perpendicular lines to these were drawn through the ventral recess of the fourth ventricle and the ventromedial part of the olive. The outer, ventral quadrant of this region (shown in yellow) became VLM, with the ventral aspect extended along the contour of the olive. Care was taken to exclude the olive nucleus from all quantitative analysis. The medial raphe (MR) region of interest was the medial quarter of the main rectangle (shown in orange), abutting the midline. This is shown for a hemi-brainstem, which was used in stereology. For whole slide scanning analysis in whole brainstem sections, identical region of interest were constructed on the opposite side and mean values over the two sides calculated. (**B**) Cresyl violet-stained neurons in the VLM/reticular formation (*top*); in the *bottom* image a neuron distended with lipofuscin is shown. (**C**) Line graphs of neuronal densities on cresyl violet stain in the VLM plotted as mean values (error bars are standard deviations) in SUDEP and control groups relative to the obex level (*x*-axis) at 2-mm intervals from 1 to 10 mm. There were significant differences between SUDEP and controls at obex 3–4 mm (asterisk). (**D**) SST labelling in a hemi-medulla section with a band of staining fanning out from ventricle to lateral reticular regions (arrow); note also labelling in the solitary nuclei. (**E** and **F**) Examples of patterns of SST labelling are shown with SST-SOMA^+^ neurons and diffuse cytoplasmic positivity (arrows indicate unstained neurons); in **G** and **H**. SST-PERIPH^+^ neurons with peripheral synaptic like beads of positivity but negative cytoplasm are shown. (**I**) Bar graph representing the fraction of total cells in VLM labelled with SST showing SST-SOMA^+^ and SST-PERIPH^+^ patterns in eight groups. SST-SOMA^+^ fractions were not significantly different between groups but significantly lower SST-PERIPH^+^ cells were noted in SUDEP (Error bars represent standard deviations for the groups; see Table 3 for significant differences in mean neuronal numbers (and standard deviations) between groups. (**J**) Line graph of variation of SST-SOMA+ neurons with obex level in SUDEP and non-epilepsy sudden death controls expressed as ratio of total neuronal densities (error bars represent standard deviations for the groups); the relative number of labelled cells increased with more rostral obex levels and the black dashed line shows values for all cases which correlated with higher obex levels (*P* = 0.014). (**K**) Line graph of variations of SST-PERIPH^+^ neurons with obex level between non epilepsy controls, non-epilepsy sudden death, SUDEP and definite-SUDEP expressed as neuronal density acquired from stereology data (mean values and error bars are standard deviations). The density of neurons declines for all groups with rostral obex levels but is lower in the epilepsy groups compared to the non-epilepsy controls at all obex levels, with the most significant differences noted between definite SUDEP and non-epilepsy sudden death controls at obex 7–8 mm (*P = 0.05). Magnifications: hemi-brainstem images taken at ×0.58 and photomicrographs with ×40 objective lens. Scale bar in **A** = 1.5 mm for **A** and **D**, and 55 μm in **B** and **E**–**H**. D-SUDEP = definite SUDEP; NEC = non-epilepsy controls; NESD = non-epilepsy sudden death controls.

Further adjacent sets of 10 interval sections, 200 µm apart, were stained for SST, NK1R, TPH2 and serotonin transporter (SERT). Single sections from the mid-obex region or each case were double labelled for NK1R/SST and TPH2/SERT and two sections from either end of the obex region under study were stained for galanin. Immunohistochemistry and double labelling immunofluorescence used standard staining protocols, detailed in the Supplementary material and in Table 2. Two regions of interest were selected for quantitation: (i) the VLM quadrant was outlined geometrically on each section using coordinates from clearly-defined anatomical landmarks of the inferior olive nuclei and the central recess of the fourth ventricle (Fig. 1A). We considered this essential as the pre-BötC does not have well defined boundaries compared to other brainstem nuclei (Schwarzacher *et al.*, 2011) and this method therefore ensured comparable capture in each case of the reticular formation, including intermediate and lateral reticular nuclei (Paxinos and Huang, 1995). (ii) The medullary raphe region of interest extended from the fourth ventricle to the olive ventrally and abutted the midline (Fig. 1A); this ensured inclusion of serotonergic neurons in both raphe obscurus and raphe pallidus (Benarroch, 2014).

**Table 2:** Immunohistochemistry panel

Immunomarker	Clone and source	Dilution	Region of interest	Quantitative method
Cresyl violet/Nissl	-	-	VLM	Stereology
Somatostatin (SST)	Rb H-106, Santacruz Biotechnology	1:500	VLM	Stereology, WSS
Neurokinin 1 receptor (NK1R)	S8305, Sigma Aldrich	1:5000	VLM	WSS
Galanin	sc-166431, Santacruz Biotechnology	1:1000	VLM, MR	WSS
Tryptophan hydroxylase (TPH2)	AB121013, Abcam	1:1500; goat polyclonal	VLM, MR	Stereology, WSS
Serotonin transporter (SERT or 5HTT)	MAB5618, Millipore	1:2500; mouse monoclonal	VLM, MR	WSS
TPH2/SERT	MAB5618, Millipore	1:500/1:2000	VLM, MR	IF, Zen
NK1R/SST	MAB5618, Millipore	1:500/1:2000	VLM	Qualitative evaluation only

These were used to assess the pre-Bötzinger region and medullary raphe, and the quantitative methods used to assess each marker is indicated. IF = immunofluorescence co-localization; MR = medullary raphe; WSS = whole-slide scanning image analysis.

### Quantitative methods and image analysis

All sections were analysed quantitatively and image analysis appropriate for the staining pattern was carried out.

#### Stereology

In cresyl violet and SST sections, total neuronal number and density were measured using the optical fractionator method with a Zeiss microscope (Axioskop 2 mot Plus) 63× objective oil emersion lens (aperture 1.4) and StereoInvestigator® software (Microbrightfield Biosciences). A sampling fraction of 5% was selected for each region of interest for cresyl violet and SST, using a counting box (100 × 100 µm and depth 10 µm) to keep the coefficient of variation low (Gunderson *P* < 0.01). Total neuronal counts in the whole rostro-caudal region of interest were estimated. In addition, neuronal densities for each section (neurons/µm^3^) in each region of interest were calculated relative to that obex level. In TPH2 sections, in view of the low density of neurons in the VLM, a modified method was used. Scanned images of the entire VLM region of interest were imported into ImageJ (NIH, USA); all positive cells were manually tagged and the cell density/area (µm^2^) calculated.

#### Whole-slide scanning image analysis

Immunostained slides were scanned with a Leica SCN400F digital slide scanner (Leica Microsystems) at 40× magnification and analysed with Definiens Tissue Studio software 3.6 (Definiens AG, Munich, Germany) using the same region of interest on both sides (Fig. 1A) and taking care to exclude any artefacts or the edges of olivary nuclei. The intensity threshold for positive labelling was set separately for each immunomarker (Supplementary Fig. 1A); the total area of staining was evaluated and expressed as a labelling index (percentage area stained) for each region of interest, averaged over both left and right sides for entire medulla sections. For SST, NK1R and galanin labelling, a second analysis step was used in view of the complex pattern of neuronal and fibre network labelling, providing two measures per case: labelling index (evaluating all thresholded positive pixels) and ‘smoothed’ labelling index (utilizing a Gaussian smoothing filter) (Supplementary Fig. 1B). As for stereology, individual values for each section with respect to the obex level were recorded in addition to the overall value. Repeatability of measurements was tested with good agreements.

#### Double labelling analysis

NK1R/SST and TPH2/SERT labelled slides were visualized using a confocal scanning laser microscopy (LSM710; Zeiss) and co-localization quantified with a Zeiss Axio Imager Z2 fluorescent microscope (Supplementary material).

### Statistical analysis and clinicopathology correlations

Statistical analysis was carried out using SPSS version 22 (IBM corporation, CA, USA) using Mann-Whitney, Kruskall-Wallis and Spearman’s correlations for non-parametric data. Data from the entire rostro-caudal region of interest were compared between groups (SUDEP, definite SUDEP, Dravet syndrome, epilepsy controls, non-epilepsy controls and non-epilepsy sudden death controls) for statistical differences with *P*-values of <0.05. In addition, differences between groups with respect to obex levels (using values averaged over five increments: obex 1–2, 3–4, 5–6, 7–8 and 9–10 mm) were evaluated to assess patterns and variations across the rostro-caudal medulla). For graphical representation of data, Graphpad Prism 7 (University of California, San Diego) was used.

## Results

### Nissl staining

Neuronal cells of varying size, morphology and lipofuscin content were present in the VLM in cresyl violet stained sections in all cases and included in the stereological analysis (Fig. 1B). Total neuronal numbers were not statistically significant differences between groups (Table 3). However, analysis of mean neuronal densities at 2 mm obex intervals showed significantly lower neuronal densities at obex 3–4 mm in all SUDEP cases compared with non-epilepsy controls (*P* = 0.008) (Fig. 1C). There were no significant differences at other obex levels or between other groups (Fig. 1C).

**Table 3:** Stereology counts on the VLM quadrant

Group classification	CV	SST SOMA+	SST PERIPH+	SST NEGATIVE	TPH2^a^
	Total neurons (SD)	Total neurons (SD)^a^^,^^b^	Total neurons (SD)^a^^,^^b^	Total neurons (SD)^a^^,^^b^	Neuronal density (×10^−6^/µm^2^)
All SUDEP	80 530 (21 673)	17 759 (6161)	15 817 (5915)	35 834 (6782)	1.3 (0.4)
		*n = *17	***P = *0.003 (NEC)**	*n = *17	
			***P = *0.01 (NESD)**		
			*n = *17		
D-SUDEP	83 499 (23 124)	17 214 (5821)	14 750 (4666)	36 548 (7529)	1.4 (0.4)
		*n = *10	***P = *0.01 (NESD)**	*n = *10	
			*n = *10		
SUDEP (excluding Dravet syndrome)	80 772 (22 709)	17 713 (6213)	15 424 (4325)	37 296 (5971)	1.4 (0.4)
		*n = *14	***P = *0.003 (NEC)**	*n = *14	
			*n = *14		
Dravet syndrome	68 567 (15 167)	17 406 (5282)	16 076 (8750)	30 319 (8403)	1.5 (0.8)
		*n = *6	***P = *0.04 (NEC)**	*n = *6	
			*n = *6		
EP-controls (excluding Dravet syndrome)	80 547 (18 647)	19 824 (3373)	18 253 (6056)	39 314 (9899)	1.4 (0.4)
		*n = *6	*n = *6	*n = *6	
All epilepsy controls	76 095 (17 837)	18 687 (3883)	16 571 (6115)	37 389 (10 816)	1.7 (0.5)
		*n = *8	***P = *0.01 (NEC)**	*n = *8	
			*n = *8		
NEC	81 011 (17 191)	14 919 (8503)	20 677 (11 380)	31 803 (16 910)	2.1 (1.3)
		*n = *11	*n = *11	*n = *11	
NESD	79 756 (19 107)	13 775 (8945)	18 988 (12 294)	28 068 (16 582)	2.0 (1.3)
		*n = *8	*n = *8	*n = *8	

This is shown for the eight group categories with mean total neuronal counts (SD) for the region of interest (Fig. 1A) equivalent to 2 mm in rostro-caudal direction.

**Figure 2:**
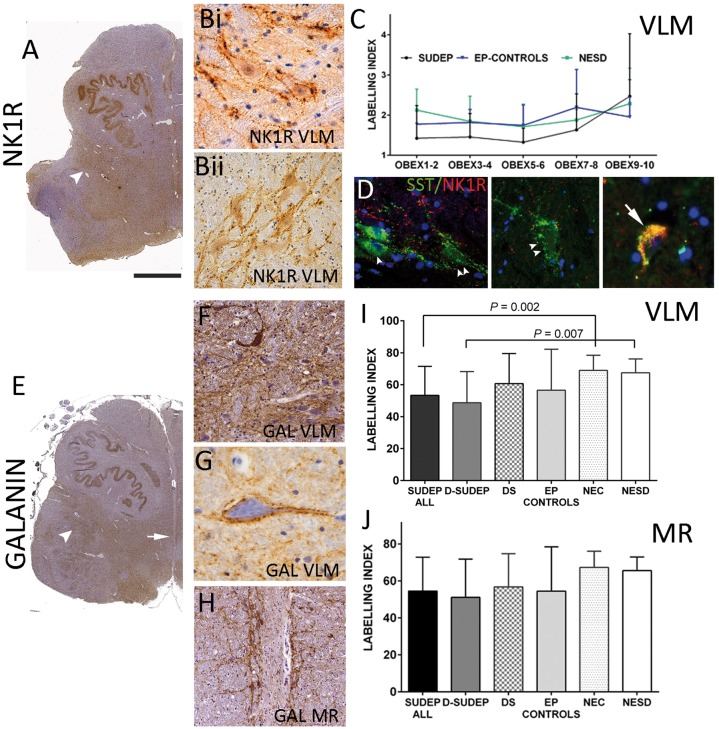
**Whole slide scanning analysis (NK1R and galanin).** (**A**). NK1R at low power showing diffuse staining in the reticular formation including a band extending into the lateral regions (arrowhead). (**Bi** and **ii**) At higher magnification complex networks and neuronal positivity with NK1R is seen as peripheral labelling around neurons. (**C**) NK1R line graph of the labelling index (smoothed) in relation to obex level between SUDEP, non-epilepsy sudden death controls (NESD) and epilepsy controls (mean values and standard deviation shown as error bars): there was no clear increase in percentage labelling with obex and lowest labelling index were noted in the SUDEP groups with significant difference between the SUDEP and non-epilepsy controls at obex 3–4 mm (*P* = 0.04). (**D**) NKR1 and SST double labelling in the VLM in different cases showing SST-SOMA^+^ (arrowhead), peripheral SST and NK1^+^ (double arrowhead) and double-labelled cells (arrow). (**E**) Galanin labelling at low magnification in a SUDEP case showing a diffuse band of labelling extending through the VLM region (arrowhead) and distinct labelling is noted around the midline raphe nuclei (arrow; shown at higher magnification in **H**). (**F**) Galanin in the VLM labelled scattered neurons but more prominent dense networks of processes and fibres and surrounding individual neurons (**G**) in the VLM was noted. (**H**) Similar intense patterns of galanin labelling were noted in the medullary raphe (MR) neuronal groups. (**I**) Bar graphs of galanin-labelling between group in the VLM and (**J**) in the medullary raphe showing significant reduction in the VLM in the SUDEP cases. Magnifications: hemi-brainstem images taken at ×0.58 and photomicrographs (**B**, **C** and **G**) with ×40 and (**F** and **H**) ×20 objective lens. Scale bar in **A** = 3 mm in **A** and **D**, 500 μm in **H**, 50 μm in **B**, **D** and **G**; and 100 μm in **F**. D-SUDEP = definite SUDEP; DS = Dravet syndrome; EP = epilepsy controls; NEC = non-epilepsy controls.

In SST sections, three neuronal cell types were counted, those with intense SST cytoplasmic labelling (SST-SOMA), neurons surrounded by a peripheral rim of synaptic labelling (SST-PERIPH) and unlabelled neurons (Fig. 2).

^a^*n* = cases available for this study.

^b^In one Dravet syndrome case and two non-epilepsy sudden death cases of the study group of 40 the staining was suboptimal and data analysis not included. Statistical differences shown in bold using the Mann-Whitney tests.

CV = cresyl violet; D-SUDEP = definite SUDEP; EP = epilepsy controls; NEC = non-epilepsy controls; NESD = non-epilepsy sudden death.

### Somatostatin

Medium-to-large neurons were labelled in the medulla with SST, being mainly dispersed through the reticular formation (Bouras *et al.*, 1987); there was no qualitative difference in labelling patterns between groups. In addition, networks of SST-positive varicose or beaded fibres and terminals were prominent in the lateral reticular region (Bouras *et al.*, 1987); these were visualized as a fan-like band of labelling at low power extending from the fourth ventricle (Fig. 1D). Distinct neuronal types were noted with SST in the VLM and were analysed separately: (i) neurons with intense SST cytoplasmic labelling (SST-SOMA^+^) (Fig. 1E and F); (ii) neurons surrounded by a peripheral rim of synaptic labelling (SST-PERIPH^+^) (Fig. 1G and H); and (iii) SST-negative neurons (Fig. 1E and F, arrows). In addition, whole-slide scanning image analysis (WSS) was used to quantify the overall labelling of cells and fibres in the VLM. The rationale of this approach was to distinguish SST-expressing neurons from SST synaptic terminals and networks (that modulate neurons) in the VLM.

SST SOMA+-labelled neurons represented from 22% (in non-epilepsy controls) to 26% (in Dravet syndrome) of all VLM neurons between the groups (Fig. 1I); total neuronal counts varied between groups but without significance (Table 3). SST-PERIPH^+^ neurons represented from 21% (in definite SUDEP) to 31% (in non-epilepsy sudden death controls) of all neurons (Fig. 1I); total neuronal counts were significantly lower in SUDEP groups compared to non-epilepsy controls and non-epilepsy sudden death controls (*P* ≤ 0.01) with less significant reductions noted for epilepsy controls and Dravet syndrome compared to non-epilepsy controls (Table 3 and Fig. 1I) (*P* < 0.05 to 0.01). There was a significant increase in the relative proportion of SST-SOMA^+^ neurons with higher mid-obex level for the region of interest (*P* = 0.014), in keeping with previous reports (Schwarzacher *et al.*, 2011) (Fig. 1J). This trend was not seen for SST-PERIPH^+^ neurons, which if anything declined in number in the rostral obex (Fig. 1K). Further analysis of mean neuronal densities at 2 mm obex increments did not show significant differences for SST-SOMA^+^ neuronal densities between SUDEP and control groups (Fig. 1J). For SST-PERIPH^+^, lower densities were noted for all epilepsy groups compared to non-epilepsy controls at all obex levels, with greatest significant at obex 7–8 mm (definite SUDEP: non-epilepsy sudden death controls) (*P* < 0.05) (Fig. 1K). WSS analysis did not show significant differences in labelling index between groups (Table 4) or in relation to obex level.

**Table 4:** Whole slide scanning analysis in VLM and medial raphe with mean values shown for all eight groups

	SST		NK1R		Galanin		TPH2		SERT	
Group classification	VLM	VLM (smoothed^a^)	VLM	VLM (smoothed^a^)	VLM (smoothed^a^)	MR (smoothed^a^)	VLM	MR	VLM	MR
All SUDEP	6.2 (1.4)	2.4 (0.9)	8 (1.7)	**1.5 (0.6)**	**53.3 (18)**	54.4 (18)	**0.7 (0.7)**	**1.7 (2)**	27.9 (14)	**40.4 (13)**
	*n = *17	*n = *17	*n = *17	***n = *17**	***n = *15**	*n = *15	***n = *17**	***n = *17**	*n* = 17	***n = *17**
D-SUDEP	6.3 (1.7)	2.6 (1.2)	8.2 (1.8)	1.6 (0.8)	**48.6 (19)**	51 (20)	**0.5 (0.3)**	**1.1 (0.9)**	34.9 (13)	**36.4 (9)**
	*n = *10	*n = *10	*n = *10	*n = *10	***n = *10**	*n = *10	***n = *10**	***n = *10**	*n = *10	***n = *10**
SUDEP (excluding DS)	6.2 (1.6)	2.6 (1)	7.7 (1.7)	**1.6 (0.7)**	**49.7 (17)**	52.7 (19)	0.7 (0.7)	1.6 (1.7)	38.1 (13)	39.5 (11)
	*n = *14	*n = *14	*n = *14	***n = *14**	***n = *12**	*n = *12	*n = *14	*n = *14	*n = *14	*n = *14
Dravet syndrome	6.1 (0.9)	2.1 (0.4)	8.9 (0.9)	1.3 (0.4)	60.6 (18)	56.8 (17)	1.3 (1.3)	2.4 (3.7)	41.2 (18)	47.9 (16.7)
	*n = *7	*n = *7	*n = *5	*n = *5	*n = *6	*n = *6	*n = *7	*n = *7	*n = *7	*n = *7
EP-controls (excluding DS)	5.2 (0.8)	2.1 (0.5)	7.7 (0.6)	1.8 (0.4)	53.2 (29)	50.6 (26)	1.8 (2.1)	1.9 (1.0)	40 (10)	42.7 (10)
	*n = *6	*n = *6	*n = *6	*n = *6	*n = *6	*n = *6	*n = *6	*n = *6	*n = *6	*n = *6
All epilepsy controls	5.6 (1.1)	2.3 (0.5)	7.9 (0.6)	1.7 (0.4)	56.5 (25.6)	54.4 (23)	1.5 (1.9)	1.7 (0.9)	42 (12)	45.3 (10.8)
	*n = *8	*n = *8	*n = *8	*n = *8	*n = *8	*n = *8	*n = *8	*n = *8	*n = *8	*n = *8
NEC	5.7 (0.9)	2.7 (1)	7.7 (2)	**2.2 (1)**	**68.9 (9.5)**	67.3 (8.7)	**2.9 (3.4)**	**5.7 (7.8)**	43.5 (20)	**52.8 (11)**
	*n = *12	*n = *12	*n = *13	***n = *13**	***n = *10**	*n = *10	***n = *12**	***n = *12**	*n = *13	***N = *11**
NESD	5.5 (0.8)	2.3 (0.3)	7.2 (2)	**1.9 (0.5)**	**67.4 (8.7)**	65.6 (7)	**2.0 (1.1)**	**3.5 (2.1)**	42 (23)	**51.9 (12)**
	*n = *10	*n = *10	*n = *10	***n = *10**	***n = *9**	*n = *9	***n = *9**	***n = *9**	*n = *10	***n = *8**

All obex levels are included in this analysis. All values are shown as labelling index [shown as percentage of area with immunostaining (i.e. range 0–100)].

*n* = the number of cases studied in each group with each marker (in occasional cases with each marker the section staining failed quality control).

^a^‘Smoothed’ data refers to additional Gaussian filters used on Definiens image analysis (see ‘Materials and methods’ section); for Galanin only the smoothed data is shown but both total and smoothed data showed significant differences between SUDEP and controls (see ‘Results’ section). Significant results highlighted in bold between SUDEP and controls (see ‘Results’ section). See also Supplementary Fig. 1 for graphs. Values in bold represent data with significant differences between SUDEP and control groups.

DS = Dravet syndrome; EP = epilepsy controls; MR = medial raphe; NEC = non-epilepsy controls; NESD = non-epilepsy sudden death controls.

### Neurokinin 1 receptor

NK1R labelling highlighted a zone extending from the floor of the fourth ventricle through the reticular formation in all groups (Fig. 2A); this corresponded on higher magnification to a plexus of processes with a mainly peri-membranous labelling of neurons (Fig. 2B). It was not possible to clearly discriminate positive from negative neurons and WSS was used for quantitative analysis rather than stereology. Lower mean NK1R labelling index (smoothed) was noted in all SUDEP cases compared to non-epilepsy controls (*P* = 0.02) and non-epilepsy sudden death controls (*P* = 0.046) (Table 4 and Supplementary Fig. 2A). There was no correlation between the NK1R labelling index and the mid-obex level of the region of interest. Further comparison of mean labelling index in cases at 2 mm obex intervals between groups showed lower labelling index in SUDEP compared to non-epilepsy controls at obex 3–4 mm (*P* = 0.04) (Fig. 2C).

#### NK1R:SST co-localization

Double labelling confirmed previous observations (Schwarzacher *et al.*, 2011; Wei *et al.*, 2012) with mixed populations of double-labelled, single-labelled cells as well as neurons with a peripheral pattern of SST labelling (Fig. 2D); consistent labelling was not achieved in all cases and further quantitative evaluation of the series was not carried out. However, we observed a strong correlation between SST and NK1R labelling index on single-stained sections (all cases *P* < 0.000, Supplementary Fig. 2B and C; SUDEP alone *P = *0.001 Supplementary Fig. 2D; epilepsy controls *P* = 0.007; non-epilepsy controls *P* = 0.001). We did not observe any relationship between the relative SST/NKR1 labelling index and obex level.

### Galanin

Labelling for galanin, both in the VLM and in the medullary raphe regions, was prominent at low magnification in all groups (Fig. 2E and H) mainly forming dense networks of processes surrounding neurons, with scattered positive neurons in the VLM (Fig. 2F) as well as some neurons with prominent pericellular labelling (Fig. 2G). WSS analysis showed significantly lower labelling index in VLM in SUDEP compared to non-epilepsy controls (*P = *0.002; for total and smoothed labelling index) and for definite SUDEP compared to non-epilepsy sudden death controls (*P* < 0.007; for total and smoothed labelling index) but not for other groups (Fig. 2I and Table 4). In the medullary raphe, less significant reductions in labelling index in SUDEP compared to non-epilepsy controls (*P* = 0.035) and definite SUDEP to non-epilepsy sudden death controls were noted (*P* = 0.04) (Fig. 2J and Table 4). There was no correlation between the galanin labelling index and obex level.

### Tryptophan hydroxylase

**Figure 3:**
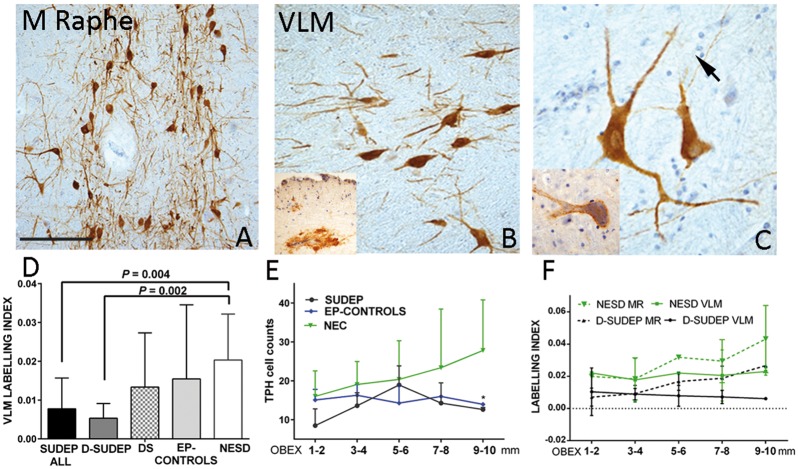
**Serotonergic neurons.** (**A**) Tryptophan hydroxylase (TPH2) labelling in the median raphe showing distinct neuronal labelling and processes. (**B**) In the VLM, reduced density of neurons were noted (inset cluster of neurons in the floor of the fourth ventricle were occasionally also noted). (**C**) TPH2-positive neurons and coarse dendrites in VLM with occasional fine axon crossing in the background (arrow). Inset: TPH2 positive neurons in VLM with more peripheral labelling pattern was occasionally noted. (**D**) Bar chart showing the differences in labelling index between the groups in the VLM, which was significantly lower in SUDEP groups than non-epilepsy controls. (**E**) Line graph of mean TPH2 cell counts between groups (mean values and standard deviation show as error bars) in the VLM with obex intervals were lower for the SUDEP and epilepsy controls than non-epilepsy controls (NEC) at all levels, with the greatest statistical difference noted between all epilepsy cases and controls at obex 9–10 mm (*P* = 0.034). (**F**) Line graph of TPH2 labelling in medullary raphe and VLM (shown as dashed lines and single lines, respectively) of mean values (and error bars representing standard deviations) with respect to obex levels for definite SUDEP and non-epilepsy sudden death controls (NESD). A positive correlation of medullary raphe labelling index with more rostral obex levels (*P* = 0.01) was noted and lower labelling index in SUDEP than NESD. Magnifications: photomicrographs with ×10 (**A**), ×20 (**B**) and ×40 objective lenses. Scale bar in **A** = 300 μm in **A**, 200 μm in **B**, and 90 μm in **C**.

Positive neurons with TPH2 were prominent in medullary raphe nuclei (corresponding to the raphe magnus, obscurus and pallidus), forming dense aggregates (Fig. 3A); a similar distribution was noted in all groups. Scattered positive cells were also present through the reticular formation and VLM region (Fig. 3B), in keeping with previous descriptions (Paxinos and Huang, 1995; Tada *et al.*, 2009; Benarroch, 2014). Immuno-labelling was primarily in the neuronal soma, extending into proximal processes, with rarer long traversing processes (Fig. 3C). In the VLM, occasional neurons were noted with accentuated peripheral labelling (Fig. 3C, inset). TPH2-positive neurons were also noted in the arcuate nuclei, single cells along the sub-pial border of the lateral medulla, and occasionally in the floor of the fourth ventricle and near the dorsal vagal nuclei.

TPH2 cell densities varied between groups but were not significantly different (Table 3) and there was no correlation between TPH2 cell counts and obex level. Comparison of mean TPH2 cell counts in the VLM at 2 mm obex intervals between groups were lower for the SUDEP and epilepsy controls than non-epilepsy controls at all levels, with statistical difference noted between all epilepsy cases and controls at obex 9–10 mm (*P* = 0.034) (Fig. 3E).

The TPH2 labelling index was consistently higher in the medullary raphe than VLM in all groups (Table 4). The TPH2 labelling index in the VLM was significantly lower in SUDEP and definite SUDEP groups than non-epilepsy control groups (both non-epilepsy controls and non-epilepsy sudden death controls) (*P* < 0.005); no significant differences were noted between Dravet syndrome and epilepsy controls compared to non-epilepsy control groups (Table 4 and Fig. 3D). For the medullary raphe, a similar reduction was noted in SUDEP groups compared to non-epilepsy controls but with less statistical significance than the VLM (*P* < 0.01) (Table 4). There was a positive correlation between the labelling index in the medullary raphe and higher obex level (*P = *0.01) but not for VLM; the higher labelling index in the medullary raphe compared to VLM was most apparent between obex 3 mm to 10 mm (Fig. 3F). Further comparison of mean labelling index at 2 mm obex intervals between the groups showed lower labelling index in SUDEP (and definite SUDEP) than non-epilepsy controls (and non-epilepsy sudden death controls) with greatest significance observed in the VLM at obex 3–4 mm and 7–8 mm (*P* < 0.05) (Fig. 3F).

### Serotonin transporter

**Figure 4:**
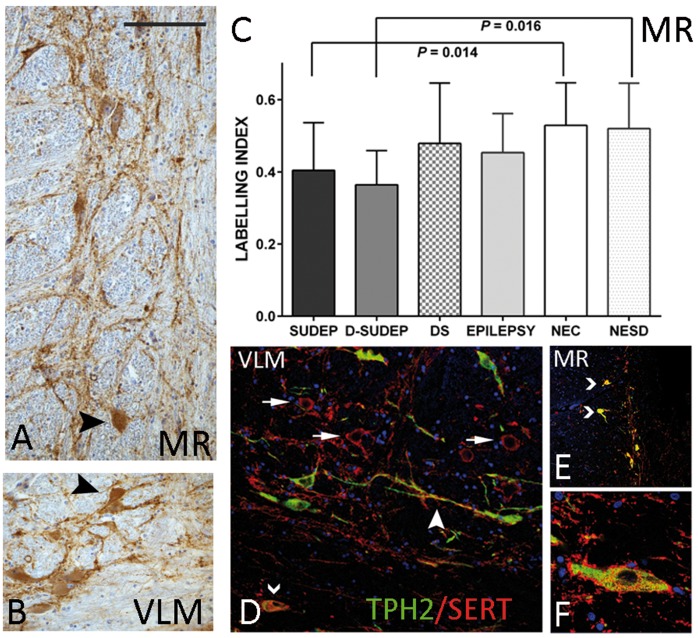
**SERT and co-localization studies.** (**A**) SERT labelling in the medial raphe (MR) and (**B**) VLM showed dense synaptic plexus of labelling mainly around neurons and processes. In addition strong cytoplasmic labelling of neuronal cells in both regions was also noted (arrowheads). (**C**) Bar chart of the mean labelling indices between groups in the medullary raphe with significant differences noted between SUDEP cases and non-epilepsy control groups. (**D**–**F**) Double labelled immunofluorescence of SERT with TPH2. There was strong regional expression of both markers concentrating in similar areas in the VLM (**D** and **F**) and medullary raphe (**E**). In the VLM, labelling of SERT around TPH2-negative neurons was evident (arrows) as well as SERT labelling at the periphery of TPH2 positive neurons (**F**) as well as processes (dendrites/axons; arrowhead in **D**). In addition, in both medullary raphe and VLM, co-localization of labelling in the cell was noted (chevrons in **F** and **E**). D-SUDEP = definite SUDEP; DS = Dravet syndrome; NEC = non-epilepsy controls; NESD = non-epilepsy sudden death controls. Magnifications: photomicrographs with ×40 objective for (**A** and **B**). Scale bar in **A** = 230 μm in **A**, **B** and **D**, 300 μm in **E** and 80 μm in **F**.

SERT immunolabelling in both the medullary raphe and VLM region consisted of a dense plexus, mainly surrounding neuronal soma and dendrites, although distinct cytoplasmic labelling of some neurons was also noted and observed in all groups (Fig. 4A and B). SERT labelling index was overall higher in the medullary raphe than VLM across all groups (Table 4). Lower SERT labelling index in the medullary raphe was noted in SUDEP than in non-epilepsy controls (*P* = 0.014) and in definite SUDEP than in non-epilepsy sudden death controls (*P* = 0.016) (Fig. 4C). There were no significant differences between the other groups in the medullary raphe or in the VLM for any groups. There was no significant correlation of SERT labelling index with obex level. Comparisons of the labelling index between groups at 2 mm obex intervals showed lower values in SUDEP, with greatest significance in the medullary raphe between SUDEP and non-epilepsy controls at obex 7–8 mm (*P* = 0.024) and in the VLM at obex 3–4 mm (*P* = 0.042).

#### TPH2/SERT co-localization

Double labelling for SERT/TPH2 showed some regional overlap in the distribution of labelling in the medullary raphe and VLM observed in all cases. SERT showed more extensive networks around processes and peripheral labelling of TPH2-positive as well as around unlabelled cells (Fig. 4D and F). In addition, several neurons in these regions showed cytoplasmic double labelling (Fig. 4D and E). Quantitative evaluation of the relative areas of co-localization was higher in non-epilepsy controls than SUDEP or epilepsy controls in both VLM and medullary raphe but were not significantly different. Co-localization was significantly higher in medullary raphe than VLM in all groups, which may merely reflect anatomical differences in serotonergic cell populations; however, greater differences were noted between these regions for SUDEP (*P* = 0.001) compared to epilepsy controls (*P* = 0.05) and non-epilepsy controls (*P* = 0.01). There was a significant positive correlation between co-localization and higher obex level for all cases for the medullary raphe (*P* = 0.009), but between groups significant correlations were noted only for the SUDEP group (VLM, *P* = 0.006 and medullary raphe, *P* < 0.000) (Supplementary Fig. 3A).

### Clinical–pathology correlations

In cresyl violet sections, there was a positive correlation between VLM neuronal density and duration of epilepsy (*P* < 0.01) and a trend for higher TPH2 neuronal counts in epilepsy patients with epilepsy duration of >10 years compared to cases with epilepsy onset <2 years (*P* ≤ 0.05, Supplementary Fig. 3B). Higher SST and NK1R labelling index was also noted in cases with epilepsy chronicity of >10 years, particularly the epilepsy control group (*P* ≤ 0.025, Supplementary Fig. 2E and F). There was no relationship between any quantified measures and gender. A positive association with the NK1R labelling index and age at death was noted (*P* < 0.01) and for TPH2, significant positive correlations were noted between age at death and labelling index in both VLM (*P* < 0.01) and medullary raphe (*P* < 0.005) (Supplementary Fig. 3C and D). We did not have complete drug histories in all cases (Supplementary Table 1), but only one person, in the control group, had been prescribed a selective serotonin re-uptake inhibitor (Case 27); SERT and TPH2 values in this case were in the middle range. There was variation in fixation times between cases collected from different centres (Table 1); we did not observe a significant correlation between labelling index or cell counts in the VLM and fixation time or relationship with post-mortem intervals.

## Discussion

There is strong clinical and experimental evidence for centrally-mediated ictal respiratory dysfunction or central apnoea as a pathomechanism in SUDEP (Ryvlin *et al.*, 2013; Sowers *et al.*, 2013; Kennedy and Seyal, 2015). MRI studies indicate structural abnormalities in the brainstem autonomic regions in SUDEP although the pathological basis is unknown (Mueller *et al.*, 2014). In a series of SUDEP post-mortem cases we have shown significant alterations in the VLM (which includes the putative human pre-BötC region) and, to a lesser degree, in the medullary raphe regions of the medulla. These involved NK1R and SST neuronal populations of the VLM. We also noted significant reductions in medullary neuromodulatory systems, including serotoninergic and galaninergic networks. There was also some evidence for an association of neuropathological alterations with duration of seizures. These findings could indicate epilepsy-mediated pathology in medullary respiratory neuronal groups that act as a vulnerability factor for SUDEP.

### SST/NK1R neuronal alterations in VLM in SUDEP

NKR1 and SST neurons in the human VLM include the putative pre-BötC, present bilaterally as an ill-defined diffuse nucleus, located near the lateral reticular and nucleus ambiguus within the intermediate reticular zone (Huang and Paxinos, 1995). Neurons of varied sizes are seen on cresyl violet stain (Paxinos and Huang, 1995; Schwarzacher *et al.*, 2011) corresponding to the mixed excitatory and inhibitory neurons in addition to the presumed ‘rythmogenic’ pacemaker subsets of NK1R/SST neurons (Bouras *et al.*, 1987; Stornetta *et al.*, 2003; Schwarzacher *et al.*, 2011; Ikeda *et al.*, 2017); from animal studies these neurons form the essential circuits that coordinate and automate inspiration (Wei *et al.*, 2012).

We noted a reduction of SST-positive neurons in the VLM specifically in SUDEP as well as a reduction of NK1R labelling, which could suggest deficient inspiratory networks. Alterations in pre-BötC NK1R and SST neurons has been previously reported in SIDS (Lavezzi and Matturri, 2008) and neurodegenerative diseases such as multiple system atrophy (Schwarzacher *et al.*, 2011) and Parkinson’s disease associated with disordered breathing (Presti *et al.*, 2014). SST is an inhibitory neuromodulator of respiration; experimental silencing of SST neurons in the pre-BötC induces apnoea (Cui *et al.*, 2016) and blocking of SST receptors prevents auto-resuscitation from asphyxia (Ramirez-Jarquin *et al.*, 2012). Slow, selective ablation of NK1R neurons results in apnoeic episodes that first occur during sleep, before ataxic breathing in wakefulness (McKay *et al.*, 2005). This implies that significant loss of pre-BötC neurons is required before pathological breathing occurs, but that the first manifestations, associated with milder loss occur during sleep (Feldman and Del Negro, 2006), which is of potential relevance to the predominance of nocturnal SUDEP cases.

Animal studies support that there is not complete overlap of expression of SST and NK1R in the pre-BötC: different neuronal subsets are present that likely reflect diverging physiological functions (Wei *et al.*, 2012; Koshiya *et al.*, 2014). Furthermore, not all neurons of a single immunophenotype share similar functions, for example not all NK1R^+^ neurons are ‘pacemaker’ cells (Ikeda *et al.*, 2017). NK1R^+^/SST-SOMA^+^, NK1R^+^/SST-PERIPH^+^ neurons (with peri-somatic SST GABAergic/inhibitory synaptic terminals) as well as NK1R^+^/SST^−^ and NK1R^−^/SST^+^ neurons have been described (Wei *et al.*, 2012), as have neurons negative for both markers. In the rodent, it is estimated that ∼15% of the 3000 pre-BötC neurons are SST-expressing (Cui *et al.*, 2016). These reports compare with our findings where 20–26% of VLM neurons were SST-SOMA^+^, and a further 21–31% were SST-PERIPH^+^, a proportion showed NK1R^+^/SST^+^ double-labelling and a strong correlation between overall NK1R and SST labelling was observed. In addition to a reduction of NK1R labelling in SUDEP, we noted specific alterations in SST neuronal populations, with a reduction in SST-PERIPH^+^, but not SST-SOMA^+^ cells or overall SST labelling. Animal studies supports that GABAergic SST^+^ terminals synapsing onto pre-BötC neurons arise from other brainstem nuclei, including the solitary tract nucleus and parabrachial nucleus (Epelbaum *et al.*, 1994; Cui *et al.*, 2016) primarily modulating respiratory activity (Cui *et al.*, 2016). Our findings could therefore be interpreted as loss of neuromodulatory SST input rather than a primary loss of SST VLM pacemaker neurons, a hypothesis that requires validation through further studies of other brainstem regions (Supplementary Fig. 4).

### Rostro-caudal distribution of NK1R and SST

Our archival cases extended over a range of obex levels and, although not different between the groups, it was important to factor in any pathological differences in relation to rostro-caudal level. Our current understanding of the connectivity and relationship of the pre-BötC with other medullary respiratory nuclear columns is based largely on animal data (reviewed in Smith *et al.*, 2013; Koshiya *et al.*, 2014; Ikeda *et al.*, 2017). Functional imaging studies of the respiratory control network in humans as yet lack the spatial resolution to distinguish individual nuclear groups (Pattinson *et al.*, 2009). In animals, there is a hierarchical organization of nuclei in a rostro-caudal direction; the Bötzinger complex lies rostral to the pre-BötC, exerting expiratory and inspiratory respiratory rhythms, respectively. Both of these nuclei are more rostral than ventral respiratory groups that coordinate output to the phrenic and spinal motor neurons (Smith *et al.*, 2013; Ikeda *et al.*, 2017). One seminal study on the human brain localized the axial level of the human homologue of the pre-BötC, based on the density of SST/NK1R neurons, between obex 6 to 14 mm and maximal at obex 9 mm; this was supported by a peak in cresyl violet neuronal densities in this region at 9 mm (Schwarzacher *et al.*, 2011). Our study, which used stereology rather than cell counting, did not show a significant variation in cresyl violet neurons in the VLM with obex level. The likely explanation for this difference is methodological, in that the region of interest we chose included the entire ventrolateral quadrant of the medulla whereas in Schwarzacher’s study the area analysed was limited to a region between the ambiguus nucleus, trigeminal tract and the inferior olive (Schwarzacher *et al.*, 2011). Other human pathology studies of pre-BötC neurons have either not defined their region of interest, used different cell counting methods or the obex level was not detailed, which precludes a meaningful comparison of data (Lavezzi and Matturri, 2008; Tada *et al.*, 2009; Presti *et al.*, 2014). Our findings of an increase in the relative proportion of SST-SOMA^+^ cells with higher obex level (obex 9–10 mm), particularly noted in controls groups, does align with Schwarzacher’s localization of the human pre-BötC (Schwarzacher *et al.*, 2011). Furthermore, the reduction in SST-PERIPH^+^ densities in SUDEP reached greatest significance at obex 7–8 mm, which may be of functional significance to inspiratory networks in the putative human pre-BötC region. The lower neuronal densities on cresyl violet and NK1R labelling in the more rostral medulla (obex 3–4 mm) in SUDEP cases could implicate pathology in more caudal ventral respiratory groups regulating motor control of respiration of equal functional importance, which requires further investigation.

### Galanergic medullary systems and relevance in SUDEP

We studied the distribution of galanin, a bioactive peptide shown to modulate brainstem serotonergic and noradrenergic systems in experimental models (Medel-Matus *et al.*, 2017) and observed reduced labelling in the SUDEP groups. Galanergic neurons in the rodent medulla are concentrated in the nucleus of the solitary tract, VLM, retrotrapezoid nucleus (Bochorishvili *et al.*, 2012; Spirovski *et al.*, 2012) and locus coeruleus (Spirovski *et al.*, 2012). In the human brainstem we noted that galanin primarily highlighted dense medullary networks, surrounding neurons but with scattered positive neurons in the VLM, in support of some local expression. The retrotrapezoid nucleus in animals, critical for central respiratory chemoreception, has glutamatergic and galanergic neurons that synapse with NK1R^+^ neurons of the pre-BötC and are considered to activate breathing (Bochorishvili *et al.*, 2012); local galanin-expressing NK1R^+^ neurons in the VLM are also activated following hypoxia and hypercapnia (Spirovski *et al.*, 2012). Other experimental studies have reported that micro-injection of galanin into the pre-BötC exerts a central respiratory depression (Abbott *et al.*, 2009) and galanin also mediates inhibition of serotonergic transmission in the medullary raphe (Medel-Matus *et al.*, 2017). Interestingly, rodent anatomical studies indicate differential sources of galanergic input to specific medullary regions; for example, there is no projection from the retrotrapezoid galanergic neurons to the medullary raphe (Bochorishvili *et al.*, 2012). Our finding of significantly reduced galanin labelling in the VLM, but not the medullary raphe, in SUDEP may be of physiological and functional relevance and warrants further in-depth investigation of human galanergic brainstem systems (Supplementary Fig. 4) and any potential influences on respiratory networks in SUDEP.

### Alterations in medullary serotonergic systems in SUDEP

The pre-BötC is further modulated by serotonergic neurons of the medulla, some of which have chemosensory properties, provide excitatory drive in conditions of hypercapnia (as reviewed in Richerson, 2004) and are mediated by several 5HT receptor subtypes (Richter *et al.*, 2003). We used labelling for TPH2, the main synthesising enzyme of 5-HT, and labelling for its presynaptic transporter (SERT) and found evidence for a reduction in the medullary serotonergic systems in SUDEP, with preferential loss of TPH2 labelling in the VLM and SERT in the medullary raphe. For TPH2 and SERT, the changes in SUDEP were maximal at higher obex levels (7–10 mm) near the putative axial level of the human homologue of the pre-BötC. In addition, co-localization of the cellular labelling of SERT and TPH2 was more significantly reduced in the VLM compared to medullary raphe in the SUDEP group. In all, these findings indicate loss of serotonergic neuronal synthesizing capacity, modified cellular re-uptake mechanisms which would result in impaired delivery of 5HT, preferentially affecting the pre-BötC region (Supplementary Fig. 4). In SUDEP, this may functionally translate as compromised auto-resuscitative responses during post-ictal hyperpcapnia.

Dysfunction of the brainstem serotonergic system in SUDEP has been proposed as a central mechanism, through effects on both ictal arousal and respiration (as reviewed in Richerson, 2013; Sowers *et al.*, 2013; Richerson *et al.*, 2016). For example, administration of serotonergic agents rescued DBA/1 mice with susceptibility to seizure-induced respiratory arrest and death (Faingold *et al.*, 2016) and both decreased firing (Zhan *et al.*, 2016) and activation (Kommajosyula *et al.*, 2017) of raphe neurons occurred following seizures. In human studies, there is a considerable literature on defective medullary serotonergic neurotransmission in SIDS. Reduced 5-HT_1A_ receptor binding in the medulla and higher densities of 5HT neurons in the medullary raphe and ventral surface were shown in SIDS but with lower 5-HTT (SERT) binding per neuron (Paterson *et al.*, 2006). We were unable to obtain reliable staining for 5-HT receptors in fixed post-mortem tissues. Possible explanations for reduced TPH2 labelling in SUDEP, compared to the findings in SIDS, could include methodological differences but also maturational effects. In our series, of mainly adults, we noted increased TPH2 labelling with age. Maturation changes to 5-HT neuronal populations have been described in infancy (Kinney *et al.*, 2011) and, furthermore in SIDS, the majority of TPH2 medullary neurons were small, immature types (Paterson *et al.*, 2006; Kinney *et al.*, 2011) compared to the large fusiform and multipolar neurons in our adult series. This highlights difficulties in the direct comparison of SIDS to SUDEP cohorts. Nevertheless, in both studies there was evidence for regional reduction in SERT labelling on TPH2 medullary neurons, which indicates a potentially common mechanism of re-uptake failure. In neurodegenerative post-mortem studies, reduction of serotonergic neurons has also been observed; loss of medullary raphe TPH2 neurons has been shown in multiple system atrophy and in Parkinson’s disease with Lewy bodies and proposed as relevant to the sleep-related disordered breathing occurring in these conditions (Presti *et al.*, 2014). Furthermore, in a study of 12 patients with multiple system atrophy, significant reduction of TPH2 neurons was shown in both the VLM and medullary raphe in those dying suddenly (Tada *et al.*, 2009).

### Clinical correlations and seizure effects on medullary neurons

The medullary neuronal changes we noted in the VLM and medullary raphe may represent acute and chronic sequelae of previous seizures. There are few pathology studies to date of the brainstem in SUDEP; in a recent study from our group, no significant differences in acute inflammatory changes in the medulla or evidence for blood–brain barrier dysfunction was observed in SUDEP compared to controls in several functionally different regions (Michalak *et al.*, 2017). There is evidence from experimental studies that seizures and ictal electrophysiological changes extend to medullary respiratory nuclei (Aiba and Noebels, 2015; Kommajosyula *et al.*, 2017; Villiere *et al.*, 2017) and could conceivably induce chronic cytopathological changes, similar to that reported in remote cortical regions, thalamus and cerebellum in human post-mortem studies (Crooks *et al.*, 2000; Blanc *et al.*, 2011; Sinjab *et al.*, 2013).

Although we did not find definitive evidence for overall neuronal loss in the VLM in SUDEP, we did note increased cresyl violet and THP2 neuronal numbers with a longer duration of seizures. Brainstem volume loss has been observed in *in vivo* MRI studies (Mueller *et al.*, 2014); any independent effect of volume changes on relative neuronal density in SUDEP requires evaluation through MRI-pathology correlative studies, which are currently in progress. There was also a trend for increased labelling with SST and NK1R in patients with epilepsy duration of a decade or more suggesting adaptive modulation of these systems can occur. There is a large literature regarding modulation of inhibitory neurons in temporal lobe epilepsy, including acquired channelopathies (Bernard *et al.*, 2004) and altered neuropeptidergic systems (de Lanerolle *et al.*, 2012) proposed to represent compensatory anti-epileptogenic mechanisms. Nevertheless, our key finding in SUDEP was of a regional reduction of medullary SST, NK1R as well as serotonergic and galanergic labelling compared to controls. We speculate this could reflect immediate consumption following a recent seizure prior to death (Ryvlin *et al.*, 2013) or accumulative depletion from recent poor seizure control (Chen *et al.*, 2017). Experimental models have shown an acute reduction of NK1R labelled neurons in the pre-BötC in the ventral respiratory column at 10 days following seizures (Totola *et al.*, 2017). Furthermore, in status epilepticus, depletion or ‘neurochemical exhaustion’ of reserves of neuropeptides, galanin and SST, and an increase in neurokinin and endocytosis of receptors, is recognized (Chen *et al.*, 2007).

In this current study, we also included a series of Dravet syndrome cases as this syndrome is associated with a higher risk of SUDEP. Dravet syndrome is considered primarily a dysfunction of GABAergic interneurons (Cheah *et al.*, 2012) and experimental studies do not yet support a primary respiratory dysfunction (Kalume, 2013). Although no distinct differences were noted compared to other groups in our study, this may relate to the small group size as well as their genetic heterogeneity.

### Limitations

In many cases only single blocks from variable obex levels were available, taken as part of the diagnostic evaluation at post-mortem examination. Furthermore, only selected neurochemical markers were examined in a limited subset of nuclei, all of which were involved in control of breathing. Because of this restricted focus, we are unable to definitively conclude that these abnormalities were the cause of SUDEP, or that SUDEP causes selective changes in these nuclei. Further studies are needed to determine whether there are much more widespread abnormalities throughout non-respiratory brainstem nuclei, and whether they are involved in the pathophysiology of these deaths. Ideally future brainstem studies in SUDEP should be conducted in prospectively sampled and clinically/genetically stratified SUDEP series with similar drug histories, ideally with fresh tissue samples. We also identified that some alterations were spatially restricted in the rostro-caudal axis; many of the differences that were detected were relatively small and may not be enough to cause a change in function severe enough to cause death. Future systematic bio-banking in SUDEP with standardized brainstem sampling will enable larger case series on aligned obex levels to further explore any rostro-caudal vulnerability of neurons systematically and their potential functional implications. Finally, the post-mortem intervals and fixation times varied between cases in our series as they were collected from various centres and brain banks in the UK. We did not find differences in staining in relation to these tissue processing times in this small series and our findings of relative differences in immunostaining between groups in different regions of interest also argue against this as having a major effect. Nevertheless, systematic biobanking in the future could overcome these potential confounding factors from variations in tissue collection and processing (Thom *et al.*, 2018).

## Conclusion

To summarize, we have demonstrated alterations in neuronal populations in the pre-BötC region of the medulla in SUDEP, with evidence for more significant alterations in neuromodulatory medullary neuropeptidergic and monoaminergic systems, including galanin, SST and serotonin. Variations noted with obex level could be relevant to differential effects within the rostro-caudal organization of respiratory nuclear groups, which requires further investigation. These alterations may represent a sequel of previous seizures and a pathological risk factor for SUDEP through defective respiratory homeostasis.

## Supplementary Material

Supplementary Data

## Abbreviations

pre-BötC: pre-Bötzinger complex
SERT/5HTT: serotonin transporter
SIDS: sudden infant death syndrome
SUDEP: sudden and unexpected death in epilepsy
VLM: ventrolateral medulla
