# Supplementary material for: The ventrolateral medulla and medullary raphe in sudden unexpected death in epilepsy
Source: Brain. 2018 Mar 28;141(6):1719–33. doi: 10.1093/brain/awy078 (PMC5972615; doi:10.1093/brain/awy078)
Supplement: Supplementary Data [file awy078_suppl_data.zip › brain-2017-01997-File010.pdf]

Supplemental Methods

CV: Briefly, the 20 µm thick formalin-fixed paraffin-embedded sections were dried overnight in a 37°C oven, followed by one hour incubation in 60°C oven, and dewaxing in xylene. Following rehydration in ethanol and water, the slides were incubated in warmed and acidified 0.01% cresyl-violet solution for 20 mins at 60°C. The slides were then washed, dehydrated in three baths of ethanol, and cleared in xylene before being cover-slipped with DEPEX mounting medium.

Immunohistochemistry (single labelling)

NK1R/ SST: The slides were dewaxed in xylene, and rehydrated in descending alcohol series. Endogenous peroxidase activity was blocked with 1% hydrogen peroxide. A heat-induced epitope retrieval method (12 minutes at 800 watts microwave) in high pH9 Tris-based buffer (NK1R), or low pH6 Citrate buffer (somatostatin) was used to unmask the antigens. The sections were blocked in 10% goat serum in PBS/T for 20 minutes at RT. This was followed by overnight incubation at 4°C with primary antibodies somatostatin 1:500 (Rb H-106, Santacruz Biotechnology), and Neurokinin 1 Receptor 1:5000 (S8305, Sigma Aldrich) in diluent buffer (Dako, UK). The next day, the sections were incubated for 30 minutes at room temperature with universal anti mouse/rabbit HRP (DAKO Envision, UK) secondary antibody. Immunocomplexes were visualized with Dako DAB+ Chromogen (Dako, Glostrup, Denmark), following which the sections were counterstained with haematoxylin, and cover-slipped. The slides were washed with phosphate-buffered saline (Fisher Scientific, Ltd., UK) between all steps.

Galanin: The slides were dewaxed in xylene, and rehydrated in descending alcohol series. Endogenous peroxidase activity was blocked with 1% hydrogen peroxide. A heat-induced epitope retrieval method (12 minutes at 800 watts microwave) in high pH9 Tris-based buffer

was used to unmask the antigens. The sections were incubated overnight at 4°C with primary antibody galanin 1:1000 (sc-166431, Santacruz Biotechnology) in diluent buffer (Dako, UK). The next day, the sections were incubated for 30 minutes at room temperature with universal anti mouse/rabbit HRP (DAKO Envision, UK) secondary antibody. Immunocomplexes were visualized with Dako DAB+ Chromogen (Dako, Glostrup, Denmark), following which the sections were counterstained with haematoxylin, and cover-slipped. The slides were washed with phosphate-buffered saline (Fisher Scientific, Ltd., UK) between all steps.

To investigate the effect of formalin-fixation on the immunolabelling (Liu, Martinian et al. 2010), immunohistochemistry was performed using all the antibodies mentioned on normal temporal neocortical tissue from blocks that have been formalin-fixed for varying intervals, ranging from 1 day to 5 months. We did not observe diminished immunostaining over this time period.

Double labelled Immunofluorescence

TPH2/SERT: Slides were incubated in 60°C oven for 2 hours, cooled to room temperature for 20 mins and dewaxed in xylene for 10 minutes. Following rehydration in 100% Industrial methylated sprits (IMS) for 2x3 minutes, slides were placed in running water for 5 minutes. Peroxidase was quenched with twenty minute incubation in 0.9% H_2O_2 and then washed in running water for 5 minutes. Slides were then placed in a Tris-based antigen retrieval solution (pH9) for SERT and acid-based (pH6) antigen retrieval solution for TPH2. Sections were microwaved for 20 minutes on high power. Subsequent to cooling in running tap water for 20 minutes, slides were placed in 1% PBS and slides were incubated with ready to use normal horse serum (2.5%) (Vector ImmPRESS Peroxidase) for 20 minutes to block any unspecific binding of the antibody. Sections were incubated with SERT (anti mouse, MAB5618, 1:2500) and TPH2 (anti goat, ab121013, 1:1500) overnight at 4°C.

The next day, slides were washed in 2x5 minutes 1% PBS. SERT slides were incubated for 30 minutes at room temperature with DAKO Real Envision HRP Rabbit/Mouse solution (DAKO K5007) while TPH sections were incubated with Vector ImmPRESS antiGoat Ig. Followed by further washes 2x5 minutes in 1% PBS, staining was visualised using DAB (DAKO K5007) solution. Counterstaining was performed with haematoxylin slides were cover slipped with Pertex. (Detailed for SERT/TPH2 with similar method for NK1R/SST)

Quantification

For quantitation of TPH2/SERT, images were captured at 20x magnification, using Axiocam MRm camera and the Zen software (Zeiss, Göttingen Germany). The area most densely stained by TPH was first selected (in the VLM and MR), and 8-15 sequential, non-overlapping fields covering the entire region (1.11 to 2.08 mm2) were captured using the same exposure times and parameters across all sections. The percentage of positive labelling with each marker and area of co-localisation were obtained using the Zen software. In three randomly-selected cases, confocal images and z stacks were acquired using confocal scanning laser microscopy (LSM710; Zeiss, Germany) to confirm co-localisation of both markers in individual neurones and processes.

Liu, J. Y., L. Martinian, M. Thom and S. M. Sisodiya (2010). "Immunolabeling recovery in archival, post-mortem, human brain tissue using modified antigen retrieval and the catalyzed signal amplification system." J Neurosci Methods **190**(1): 49-56.

Supplementary Figure 1: Method for whole slide scanning image analysis and detection using Definiens software.

Top two rows : TPH2 (Trytophan hydroxylase) detection. Immunostained slides were scanned with a Leica SCN400F digital slide scanner (Leica Microsystems, Wetzlar, Germany) at 40x magnification and analysed with Definiens Tissue Studio software 3.6 (Definiens AG, Munich, Germany). The threshold for intensity was set separately for each immunomarker but kept constant across case series. Initially the DAB staining was detected and classified into high, medium and low as shown for TPH2 staining: high (red), medium (orange) and low (yellow). For this marker, medium and high labelling was selected for analysis as representative of positive labelling. Separate thresholds set for haematoxylin staining which allowed identification and exclusion of nuclei and also artefacts are excluded from the analysis e.g. breaks in the section. The total area of staining is then evaluated and expressed as a labelling index (LI) (percentage of whole region of interest stained).

Bottom row: Galanin detection. Analysis as for the above methods with the first image showing the Definiens thresholding as raw data of all detected pixels shown as one colour (red) .The ‘smoothed’ analysis with the additional step added by application of a Gaussian filter which eliminates all positive pixels not in continuity with an adjacent positive pixel, smoothing the overall detection.

Supplementary Figure 2. NK1R and Somatostatin (SST) and clinical correlations

A. Neurokinin 1receptor (NK1R) : Bar graph of mean values (error bars represent standard deviations) from whole slide scanning and labelling index in the eight groups studied of the

ventrolateral medulla (VLM). Asterisks indicate significant differences between SUDEP and non-epilepsy controls groups (p <0.05) as the only statistical finding.

B. Scatter graphs: significant correlation between NK1R and SST labelling for all cases p<0.0000. This correlation was also significant with LI ‘smoothed’ data ((p=0.01); data not shown). (Dashed lines on B, C and D represent 95% confidence intervals)

C. Same graph as in B but colour coded for study group showing cases were not clustered into groups . D. Correlation between NK1R and SST labelling is also seen in SUDEP cases alone (p=0.01) as well as epilepsy controls (p=0.07) and non-epilepsy controls (p=0.01) (not shown in individual graphs).

E. Bar graph of SST with epilepsy chronicity : Higher labelling index was noted with chronic epilepsy of > 10 years duration compared to onset of epilepsy in 2 years prior to death; this approached significance in epilepsy control group but not SUDEP (p=0.025 and p=0.05 respectively for the LI ‘smoothed’) F. Bar graph of NK1R with epilepsy chronicity : Higher labelling index was noted with chronic epilepsy of > 10 years duration compared to onset of epilepsy in 2 years prior to death; this was significant in epilepsy controls (p=0.02) but not SUDEP cases.

SUDEP= sudden and unexpected death in epilepsy, D-SUDEP = definite SUDEP, Epilepsy controls = epilepsy controls, NESD = non-epilepsy sudden death controls, NEC= non epilepsy controls. DS = Dravet syndrome,

Supplementary Figure 3 Tryptophan hydroxylase (TPH2), serotonin transporter (SERT) and clinical correlations.

A. Line graph of relative area of co-localisation in the median raphe (MR) and obex level. There was a significant positive correlation between co-localisation and higher obex level for all cases ($p=0.009$), but between groups significant correlations were noted only for SUDEP group (MR $p<0.000$). B. Bar graph of TPH2 mean cell densities in the ventrolateral medulla (VLM) with seizure chronicity with higher cell densities with chronic epilepsy of greater than 10 years duration compared to onset of epilepsy in 2 years prior to death; this approached significance in epilepsy controls ($p=0.05$) but not SUDEP cases (error bars represent standard deviation). C. Significant correlations were noted between TPH2 labelling and age at death in the VLM and also D. in the MR (dashed lines indicate 95% confidence intervals). SUDEP= sudden and unexpected death in epilepsy, Epilepsy controls = epilepsy controls, NEC= non epilepsy controls.

Supplementary Figure 4. Schematic diagram summarising the key findings.

Schematic diagram to summarise the main alterations observed in the ventro lateral medulla (VLM), the putative human site of the pre-Botzinger complex, its neuronal populations and interactions with incoming neuromodulators in SUDEP is shown on the right side compared to control brainstem on the left. Dashed line indicates reduced labelling interpreted as compromised function of the specified modulatory system. Filled shape indicates cytoplasmic/cell expression and outline colour of shape indicates peripheral or peri-somatic labelling.

ST/SolN = solitary tract and nucleus, RTN = retrotrapezoid nucleus, MR, medullary raphe, IO= inferior olive, DV = dorsal vagus nucleus, PYR = pyramidal tracts.

Supplementary Figure 5. Individual channels for the confocal images.

Individual channels for the confocal images for co-localisation studies between SERT and TPH2 in the median raphe and VLM as shown in Figure 4 D to F (top row Figure 4D, middle row Figure 4E and bottom row figure 4F).
