# Supplementary material for: The ventrolateral medulla and medullary raphe in sudden unexpected death in epilepsy
Source: Brain. 2018 Mar 28;141(6):1719–33. doi: 10.1093/brain/awy078 (PMC5972615; doi:10.1093/brain/awy078)
Supplement: Supplementary Data [file awy078_suppl_data.zip › brain-2017-01997-File011.pdf]

GROUPS	CASE NUMBER	CAUSE OF DEATH~	AGE AT SEIZURE ONSET (YEARS)	AGE AT DEATH/GENDER	SEIZURE TYPES/CONTROL	MEDICATIONS (CURRENT AT TIME OF DEATH)	CIRCUMSTANCES OF DEATH/RELEVENT CLINICAL HISTORY/GENETIC DATA	MAIN PM FINDINGS	NEUROPATHOLOGY-MACRO	NEUROPATHOLOGY MICRO	Brain weight for SPSS Fresh (or fixed -22g)
SUDEP GROUP	1	SUDEP-DEFINITE ~	UK	42/F	1-2 SEIZURES PER YEAR	INA	FOUND DEAD ON FLOOR 14.45PM ; LAST SEEN ALIVE 11AM	PULMONARY OEDEMA	NORMAL	NORMAL	1220
	2	SUDEP-DEFINITE ~	15	33/M	SEIZURES WELL CONTROLLED	LEVTIRACETAM, TOPIRAMATE	FOUND ON FLOOR IN BEDROOM/PREVIOUS HISTORY OF CRANIOTOMY FOR BRAIN TUMOUR	PULMONARY OEDEMA	ABSENT LEFT TEMPORAL POLE	EOSINOPHILIC NEURONES CA1/ NO RESIDUAL TUMOUR	1482
	3	SUDEP-DEFINITE ~	INA	40/F	INA	INA	FOUND IN BED / THREE SEZIURES IN PREVIOUS 24 HOURS DI GEORGE SYNDROME	PULMONARY ODEMA AND ?BRONCHOPNEUMONIA	HIPPOCAMPAL ATROPHY (BILATERAL) AND ROTATIONAL ABNORMALITY	HIPPOCAMPAL SCLEROSIS ; HIPPOCAMPAL MALROTATION;	1121
	4	SUDEP-PROBABLE	INA	53/M	INA	INA	FOUND DEAD ON FLOOR /EPILEPSY, RENAL DIALYSIS AND ARTHRITIS.	MENINGIOMA ; NO CAUSE OF DEATH ASCERTAINED AT GENERAL AUTOPSY	MENINGIOMA NOTED	ANI HIPPOCAMPUS	1609
	5	SUDEP-POSSIBLE	39	40/M	GENERALISED SEIZURES	NO INFORMATION. ONLY VALPROATE DETECTED AT PM	FOUND DEAD IN BATH HISTORYOF TBI, PSYCHIATRIC DISEASE FOR 8 YEARS, GLIOMA DIAGNOSED 1 YEAR PRIOR TO DEATH BUT NOT OPERATED	NO EVIDENCE OF DROWNING MILD PULMONARY OEDEMA	OLD FRONTO-BASAL CONTUSIONS ; OLIGODENDRO-GLIOMA (GRADE II) WITH EARLY ANAPLASIA	GLIOMA ; NO HS ; OLD TBI	1476
	6	SUDEP-DEFINITE	16	18/F	GENERALISED SEIZURES	LAMOTRIGINE ; OXCARBAZINE; DIAZEPAM	NOCTURNAL DEATH FOUND FACE DOWN ON BED ;	PULMONARY CONGESTION	TUMOUR TEMPORAL LOBE ; HIPMAL;	GANGLIOGLIOMA	1310
	7	SUDEP-PROBABLE	INA	34/M	INA ON SEIZURE TYPES / 'NOT WELL CONTROLLED'	NON COMPLIENT WITH AED (NO TOXICOLOGY AT PM)	SEIZURE PREVIOUS DAY ; FOUND DEAD IN TOILET	NEGATIVE PM	NORMAL	ANI IN HIPPOCAMPUS ; GLIOSIS IN AMYGDALA	1354
	8	SUDEP - DEFINITE	'LONG-TERM'	33/F	EPIELPSY SYNDROME FROM AVAILBLE MEDICAL RECORDS	INA	FOUND DEAD ON BED	NEGATIVE GENERAL PM	NORMAL	ANI CORTEX, BG AND HIPPOCAMPI	1350
	9	SUDEP-PROBABLE	4	17/F	NO DETAILS ON CURRENT SEIZURE TYPES / FEBRILE CONVULSIONS AS CHILD	INA	FOUND DEAD IN BED, FACE DOWN. NO TONGUE BITING OR INCONTINENCE.		MILD SWELLING AND UNCAL GROVING		1449

	10	SUDEP - DEFINITE	12	42/M	NO DETAIL FOR TYPES OF SEIZURES	TOPIRAMATE, LEVERTIRACETAM, LOPERAMIDE	FOUND AT HOME ; BODY PRONE		OLD SURGICAL SCAR FROM TREATED BRAIN ABSCESS IDENTIFIED WHICH WAS FOLLOWED BY EPILEPSY	AREA OF GLIOSIS IN REGION OF OLD ABSCESS; ANI ; CEREBELLAR GLIOSIS	1333
	11	SUDEP- DEFINITE	5	44/F	NO DETAIL ON SEZIURE TYPES	NO DETAILS	FOUND LYING FACE DOWN IN BED. LAST SPOKEN TO THE EVENING BEFORE (13.5 HOURS PREVIOUSLY).		SWELLING AND UNCAL GROVING ;	ANI IN CORTEX	1384
	12	SUDEP- DEFINITE	5	33/M	PARTIAL SEZURES	CARBEMAZEPINE (DIAZEPAM TRACE DETECTED AT PM)	UNWITNESSED NOCTURNAL DEATH FOUND LYING FACE DOWN/ LIFELONG EPIELPSY AND LEARNING DISABILITY	NO CAUSE OF DEATH AT PM ; PULMONARY OEDEMA	NO SIGNIFICANT ABNORMALITY	ANI IN HIPPOCAMPUS	1465
	13	SUDEP- PROBABLE	0.9	40/M	MULTIPLE TYPES : PARTIAL AND GENERALISED SEIZURES AND DROP ATTACKS	EPILIM, LACOSAMIDE, OXCARBAZEPINE,	NO DETAILS ON CIRCUMSTANCES OF DEATH. SUDDEN DEATH. EPILEPSY FOLLLOWED VACCINATION WITH MENINGOENCEPHLAITIS	NO PM REPORT	SWISS CHEESE ARTFECT	GRANULAR EPENDYMITIS (CHRONIC)	1410
	14	SUDEP- DEFINITE	26	27/M	NOCTURNAL SEIZURE	NONE	NOCTURNAL UNWITNESSED DEATH / UNDER INVESTIGATIONS FOR SEZIURES	PULMONARY CONGESTION	FULL BRAIN	MILD HIPMAL ; ANI HIPPOCAMPUS;	1623
DRAVET SYNDROME	15	ENCEPHALOP ATHY	0.4	46/F	GENERALISED AND PARTIAL SEIZURES/ 3-4 SZ PER WEEK		DRAVET SYNDROME/ LEARNING DIFFICULTIES	NO CARDIAC ABNORMALITY	NONE	MILD CEREBELLAR ATROPHY ; VACUOLAR CHANGE IN SPINAL CORD	1125
	16	SUDEP- PROBABLE	1.5	47/M	GENERALISED, PARTIAL SEIZURES AND FEBRILE SEIZURES	VIGABATRINE CLOBAZAM CARBAMEZEPINE	SUDDEN DEATH NO DETAILS/DRAVET SYNDROME REFRACTORY EPILEPSY	NO PM REPORT AVAILABLE	LESION IN AMYGDALA	TELANGIECTASIA IN AMYGDALA ; MILD CEREBELLAR ATROPHY	1078
	17	SUDEP - PROBABLE	0.45	1/M	MYOCLONIC AND GENERALISED SEIZURES	NOT GIVEN	NOCTURNAL DEATH NOT WITNESSED /DRAVET SYNDROME; WITH SCN1A WHOLE GENE DELETION, DEVELOPMENTAL DELAY	CONGESTION ALL ORGANS (MILD) L	NORMAL ; 'SWOLLEN' BRAIN REPORTED AT pm	MILD END FOLIUM GLIOSIS	1251
	18	SUDEP- DEFINITE	0.6	11/M	GENERALISED, PARTIAL AND MYOCLONIC SZ STATUS EPILEPTICUS.	TREATED WITH VARIED AED, VAGAL NERVE STIMULATOR,	FOUND UNCONCIOUS, SEIZURE NOT WITNESSES, TREATMENT WITHDRAWN AFTER 1 DAY ON ITU/ DS WITH SCN1A SPLICE SITE MUTATION), DEVELOPMENTAL REGRESSION AND AUTISM INTELLECTUAL DISABILITY, GAIT DISORDER		SWOLLEN BRAIN, CONGESTION, TONSILLAR HERNIATION, UNCAL COMPRESSION	DIFFUSE ANI, MILD CEREBELLAR ATROPHY	1300
	19	NO DETAIL	0.16	10/F	FEBRILE, MYOCLONIC, PARTIAL, ABSENCE, GENERALISED AND STATUS EPILEPTICUS	NO DETAILS	DRAVET SYNDROME; DEVELOPMENTAL REGRESSION, SEVERE INTELLECTUAL IMPAIRMENT, AUTISM, ATAXIS. SPILICE SITE SCN1A MUTATION	NO PM REPORT AVAILABLE	NORMAL	SUBPIAL GLIOSIS ;	1040

	20	NO DETAIL	0.8	11/F	HEMICLONIC FEBRILE, ABSENCE, GENERALISED, PARTIAL, HEMICLONIC AND STATUS EPILEPTICUS.	NO DETAILS	DRAVET SYNDROME; ATAXIA, INTELLECT IMPAIRMENTS (SEVERE), BEHAVIOURAL PROLBLEMS. NO SCN1A MUTATION	NO PM REPORT AVAILABLE	NO REPORT	NO REPORT	
	21	STATUS EPILEPTICUS	1.5	5/M	NOCTURNAL SEIZURES, STATUS EPILEPTICUS, FEBRILE, GENERALISED AND PARTIAL SEIZURES.	VALPROATE, DIPHENOXYLATE HYDROCHLORIDE ATROPINE SULPHATE	STATUS EPIELEPTICUS, SEPSIS AND MUTI-ORGAN FAILURE/ DRAVET SYNDROME MISSENSE MUTATION IN SCN1A NORMAL DEVELOPMENT	LUNG CONGESTION, FATTY CHANGE IN LIVER (MILD)	CONGESTION, UNCAL GROVING , NO TONSILLAR NECROSIS	WIDESPREAD ANI ; SPARING OF CA1	1340
NON EPILEPSY CONTROLS	22	UNASCER-TAINED		40/M	NO SEIZURES	GAVISCON AND IBUPROFEN	COLLAPSED WITH CARDIAC ARREST FOLLOWING PERFORATED PEPTIC ULCER/ SCHIZOPHRENIA BUT NO SEIZURE HISTORY		NORMAL	NORMAL	1431
	23	UNASCER-TAINED		80/F	NO SEIZURES	NO MEDICATIONS / NO PMH	RECENT ONSET SEZIURES-LIKE MOVEMENTS FOR 3 DAYS PRIOR TO DEATH (NO EEG DONE) / ?ENCEPHALITIS (NO EPIELPSY HISTORY)	NEUROPATHOLOGY - NEGATIVE	NO ABNORMALITIES/ENCEPHALITIS NOT CONFIRMED	ANI IN HIPPOCAMPI ; BRAAK STAGE II ; NO ENCEPHALITIS ; NO INFARCT	1374
	24	SUDDEN CARDIAC DEATH WITH MORPHOLOGICALLY NORMAL HEART (SUDDEN ADULT DEATH / SAD)		23/M	NO SEIZURES	NO MEDICATIONS/ NO PMH	FOUND LYING ON BACK IN BEDROOM – NO EPILEPSY HISTORY	PULMONARY CONGESTION	NORMAL	ANI IN HIPPOCAMPUS	1306
	25	CORONARY ARTERY THROMBOSIS		43/M	NO SEIZURES	NO MEDICATIONS	MRC SD BRAIN BANK MATERIAL	MRC SD BRAIN BANK MATERIAL	MRC SD BRAIN BANK MATERIAL		1560
	26	BRONCHIAL ASTHMA		40/F	NO SEIZURES	ASTHAM INHALERS (NOT FOR LAST 6 MONTHS)	MRC SD BRAIN BANK MATERIAL	MRC SD BRAIN BANK MATERIAL	MRC SD BRAIN BANK MATERIAL		1260
	27	CORONARY ARTERY THROMBOSIS ,		45/M	NO SEIZURES	CITALOPRAM ASPIRIN,GTN, ATORVASTIN, BISOPROLOL, RAMPIRIL, OMEPRAZOLECOEDINE, OMACOR	MRC SD BRAIN BANK MATERIAL	MRC SD BRAIN BANK MATERIAL	MRC SD BRAIN BANK MATERIAL		1650
	28	CORONARY ARTERY ATHEROSCLEROSIS		45/F	NO SEIZURES	NO MEDICATIONS	MRC SD BRAIN BANK MATERIAL	MRC SD BRAIN BANK MATERIAL	MRC SD BRAIN BANK MATERIAL		1330

	29	BRONCHOPNEUMONIA AND CORONARY ARTERY ATHEROSCLEROSIS		44/M	NO SEIZURES	DICLOFENAC, SALBUTAMOL	MRC SD BRAIN BANK MATERIAL	MRC SD BRAIN BANK MATERIAL	MRC SD BRAIN BANK MATERIAL		1600
	30	SUSPENSION BY LIGATURE		39/M	NO SEIZURES	NIFEDIPINE	MRC SD BRAIN BANK MATERIAL	MRC SD BRAIN BANK MATERIAL	MRC SD BRAIN BANK MATERIAL		1360
	31	CARDIAC ARRHYTHMIA, PRESUMED DRUG TOXICITY		36/M	NO SEIZURES	ZOLPIDEM, OLANZAPINE, AMISULPRIDECHLORPORMAZINE	MRC SD BRAIN BANK MATERIAL	MRC SD BRAIN BANK MATERIAL	MRC SD BRAIN BANK MATERIAL		1600
	32	SUDDEN CARDIAC DEATH		43/M	NO SEIZURES	NO MEDS	MRC SD BRAIN BANK MATERIAL	MRC SD BRAIN BANK MATERIAL	MRC SD BRAIN BANK MATERIAL		1510
	33	NO COD – LIMITED PM		33/M	NO SEIZURES	DEXAMENTHASONE, LEVTIRACETAM	CEREBRAL ASTROCYTOMA – OPERATED PREVIOUS YEAR. NO SEIZURES	PM LIMITED TO HEAD	BRAIN SWELLING AND TUMOUR	CONFIRMED RESIDUAL TUMOUR ;BRAINSTEM NORMAL	1602
	34	HYPERTROPHIC CARDIOMYOPATHY		29/M	NO SEIZURES	NONE	HISTORY OF CARDIOMYOPATHY ; PASSED AWAY WHILE SITTING IN CHAIR	HYPERTROPHIC CARDIOMYOPATHY	NORMAL	FOCAL HIPPOCAMPAL ANI	1516
EPILEPSY CONTROLS	35	HEAD ONLY PM	70	72/M	PARTIAL AND GENERALISED	METRONIDAZOLE, ACYCLOVIR	CLL, PROSTATE CANCER ; SEIZURES ; SUSPECTED ENCEPHALITIS<br>RECENT ONSET OF SEIZURES	CHRONIC ENCEPHALITIS (?AUTOIMMUNE)/CLL	LAMINAR NECROSIS OF HIPPOCAMPUS AND CORTEX	LYMPHOCYTIC ENCEPHALITIS.	NOT RECORDED ON PM REPORT
	36	NO PM REPORT	INA	51/M	NO DETAILS OF TYPES	ON AED ; NO DETAIL	ON AED FOR SEIZURES BUT WELL CONTROLLED	NO PM REPORT	FULL BRAIN ; TUMOUR IN CINGULATE GYRUS, NO HERNIATION	GLIOBLASTOMA	1490
	37	SEPSIS, UTI, PANCREATITIS	13	47/M	MYOCLONIC AND GENERALISED	PIRACETAM, PRIMIDONE, CLONAZEPAM, VALPROATE,	PROGRESSIVE MYOCLONIC EPIELPSY (GENETIC PME TYPE 1 UNVERRICHT LUNDBORG DISEASE ; PNEUMONIA ; SISTER HAD PME; MRI NORMAL	PANCREATITIS	MILD CEREBELLAR ATROPHY	MILD CEREBELLAR ATROPHY ; VACUOLAR CHANGE IN SPINAL CORD WHITE MATTER	1260
	38	CCF, RENAL FAILURE	11	73/M	TYPE NOT STATED BUT LIFE LONG SEIZURES	PHENYTOIN	SEIZURES FOLLOWING MEASLES	LIMITED TO HEAD ONLY	MILD ATROPHY	POSSIBLE HETEROTOPIA	1185
	39	GASTRIC ASPIRATION ; MEGACOLON	0.3	79/M	TEMPORAL LOBE EPILEPSY	PHENOBARBITONE, EPINUTIN	LIFELONG EPILEPSY, LEFT TEMPORAL LESION CHALFONT RESIDENT ; WELL CONTROLLED	HEAD ONLY	HIPPOCAMPAL ASYMTERY AND SMALL LACUNE INFARCT	LEFT HIPPOCAMPAL SCLEROSIS : INCREASED TAU ACCUMULATION NOTED	1380

	40	HEAD ONLY PM ; COD NOT CONFIRMED	84	84/F	PARTIAL AND GENERALISED SEIZURES		ONE MONTH HISTORY UTI, SEPSIS? CVA	HEAD ONLY PM	MILD TEMPORAL AND HIPPOCAMPAL ATROPHY	WM VASCULAR DISEASE, OLD CONTUSIONS, LEWY BODY DISEASE	1223

SupplementaryTable 1. Further information on cases in study.

ANI = acute (eosinophilic neuronal injury), SUDEP = sudden and unexpected death in epilepsy, INA = information not available, TBI= Traumatic brain injury, WM = white matter, PM= post-mortem. ~ COD as provided from Coroners' inquest where done/available or based on final post-mortem report.
