# Supplementary figures and images for: The ventrolateral medulla and medullary raphe in sudden unexpected death in epilepsy
Source: Brain. 2018 Mar 28;141(6):1719–33. doi: 10.1093/brain/awy078 (PMC5972615; doi:10.1093/brain/awy078)

IHC

DEFINIENS

DEFINIENS

MR/TPH2

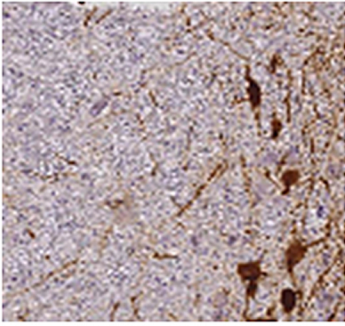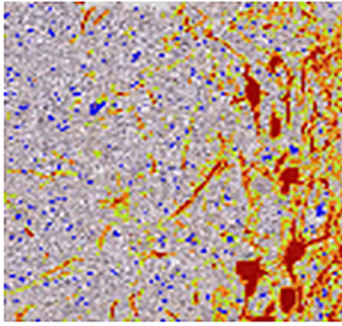

VLM/TPH2

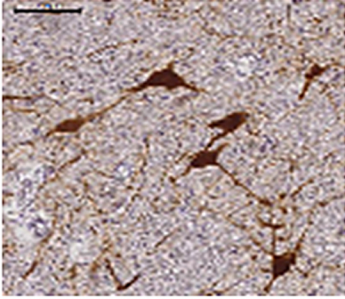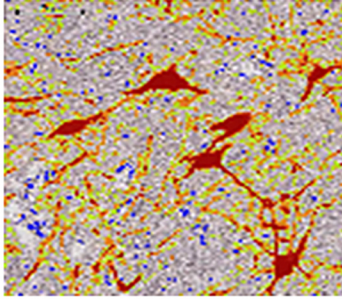

VLM/GALANIN

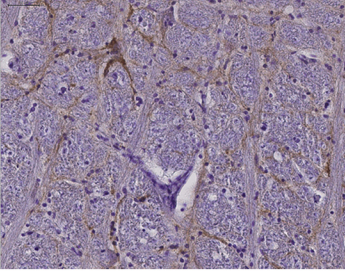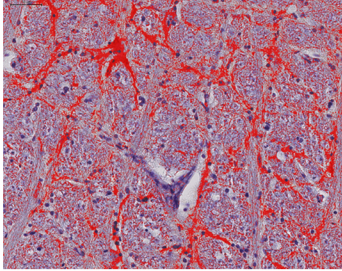

'SMOOTHED'

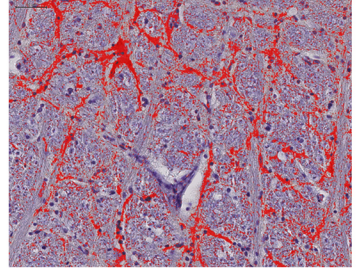

Supplement: Supplementary Data [file awy078_suppl_data.zip › brain-2017-01997-File012.pdf]

## NK1R VLM

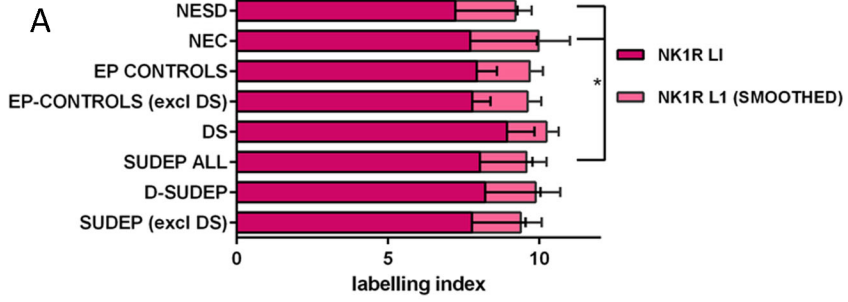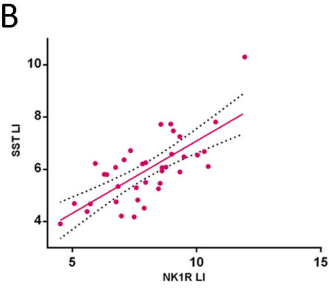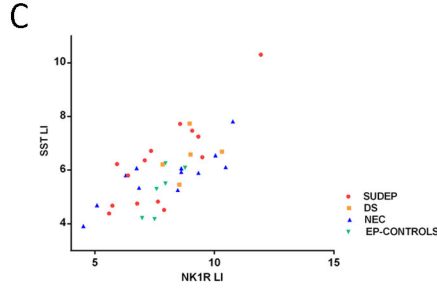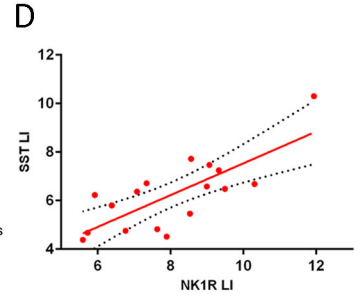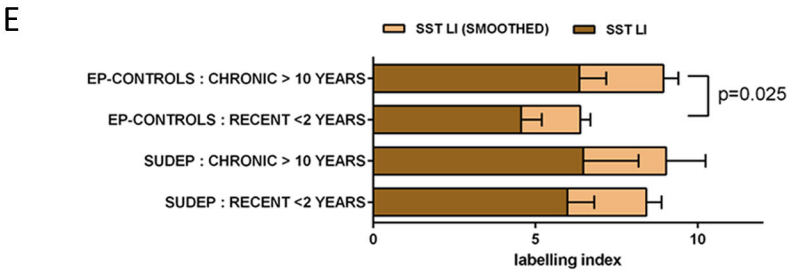

SST VLM

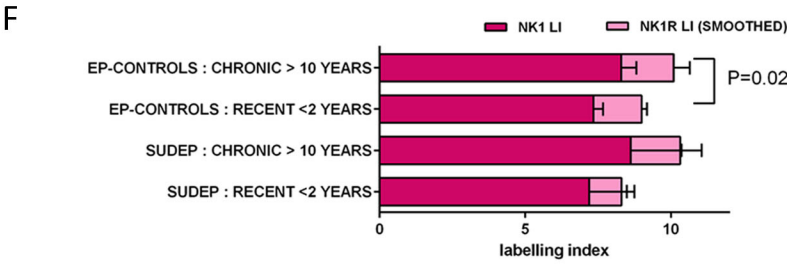

NK1R VLM

Supplement: Supplementary Data [file awy078_suppl_data.zip › brain-2017-01997-File013.pdf]

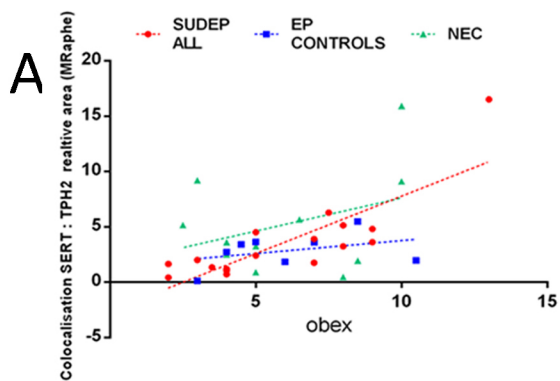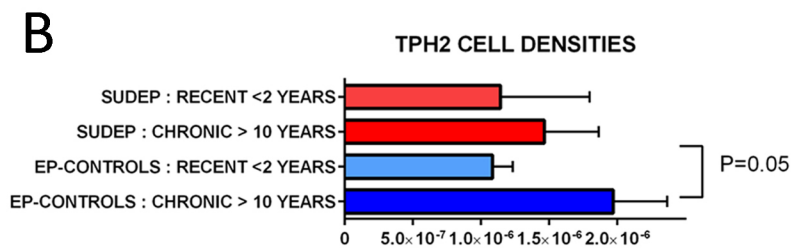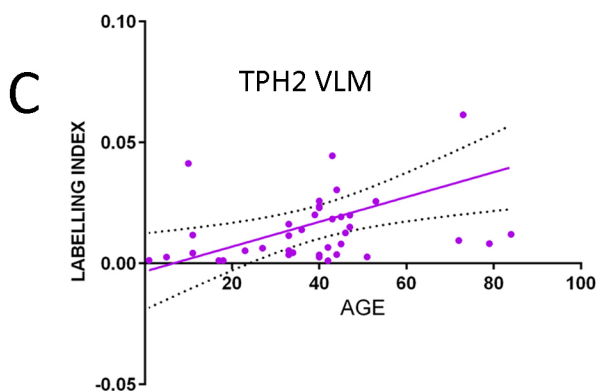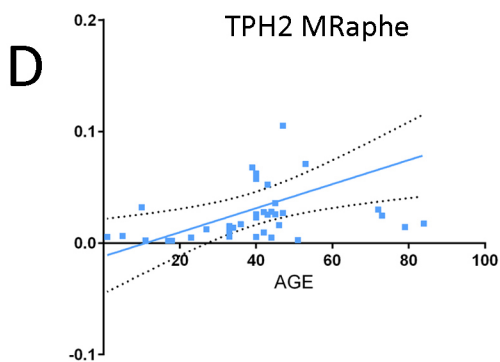

Supplement: Supplementary Data [file awy078_suppl_data.zip › brain-2017-01997-File014.pdf]

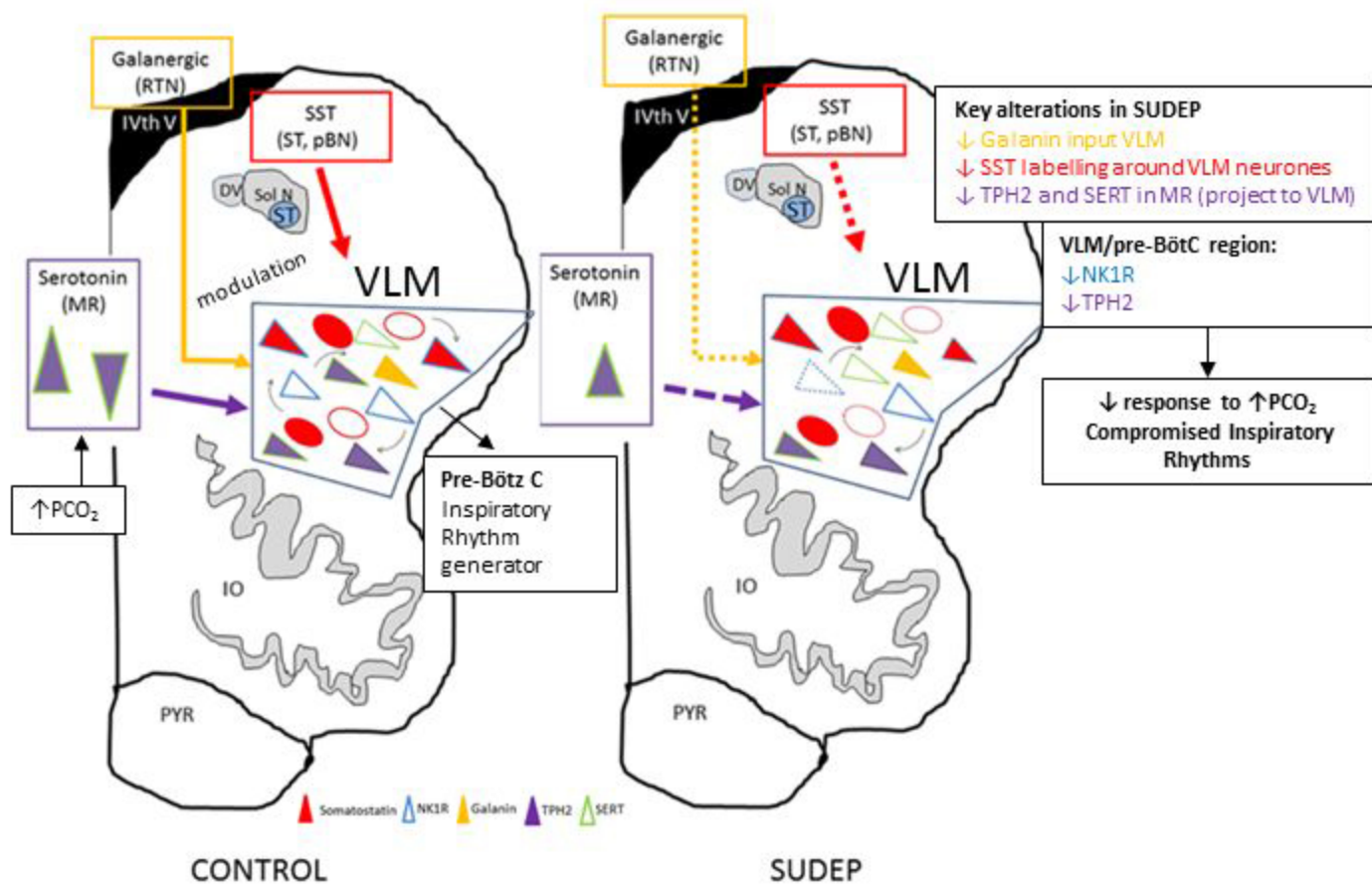

Supplement: Supplementary Data [file awy078_suppl_data.zip › brain-2017-01997-File015.pdf]

SERT

TPH2

MERGED (+DAPI)

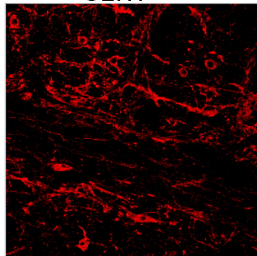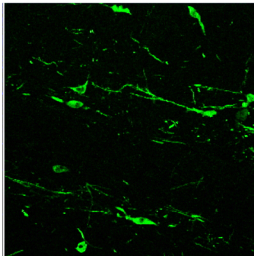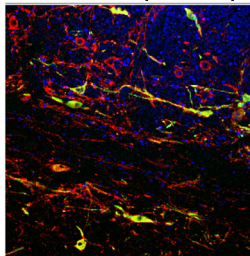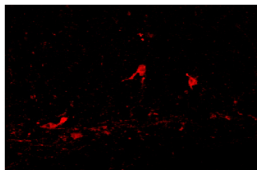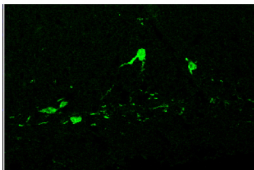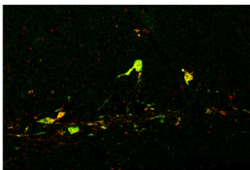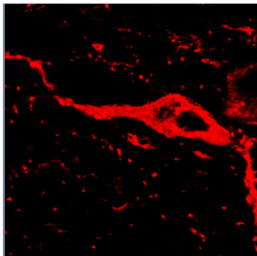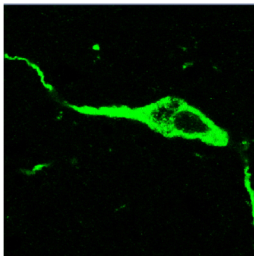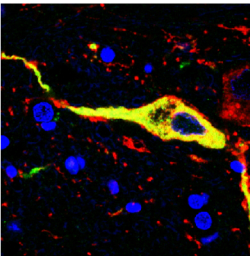

Supplement: Supplementary Data [file awy078_suppl_data.zip › brain-2017-01997-File016.pdf]
